# The *Epichloë festucae* Antifungal Protein *Efe*-AfpA Protects Creeping Bentgrass (*Agrostis stolonifera*) from the Plant Pathogen *Clarireedia jacksonii*, the Causal Agent of Dollar Spot Disease

**DOI:** 10.3390/jof8101097

**Published:** 2022-10-18

**Authors:** Patrick A. Fardella, Zipeng Tian, Bruce B. Clarke, Faith C. Belanger

**Affiliations:** Department of Plant Biology, Rutgers University, New Brunswick, NJ 08901, USA

**Keywords:** antifungal protein, fungal endophyte, plant–microbe interaction, symbiosis

## Abstract

Dollar spot disease, caused by the fungal pathogen *Clarireedia jacksonii*, is a major problem in many turfgrass species, particularly creeping bentgrass (*Agrostis stolonifera*). It is well-established that strong creeping red fescue (*Festuca rubra* subsp. *rubra*) exhibits good dollar spot resistance when infected by the fungal endophyte *Epichloë* *festucae*. This endophyte-mediated disease resistance is unique to the fine fescues and has not been observed in other grass species infected with other *Epichloë* spp. The mechanism underlying the unique endophyte-mediated disease resistance in strong creeping red fescue has not yet been established. We pursued the possibility that it may be due to the presence of an abundant secreted antifungal protein produced by *E. festucae*. Here, we compare the activity of the antifungal protein expressed in *Escherichia coli*, *Pichia pastoris*, and *Penicillium chrysogenum*. Active protein was recovered from all systems, with the best activity being from *Pe. chrysogenum*. In greenhouse assays, topical application of the purified antifungal protein to creeping bentgrass and endophyte-free strong creeping red fescue protected the plants from developing severe symptoms caused by *C. jacksonii*. These results support the hypothesis that *Efe*-AfpA is a major contributor to the dollar spot resistance observed with *E. festucae*-infected strong creeping red fescue in the field, and that this protein could be developed as an alternative or complement to fungicides for the management of this disease on turfgrasses.

## 1. Introduction

Antifungal proteins have been reported from several fungal species [1,2]. The most well-characterized are those from *Penicillium chrysogenum*, designated PAF and PAFB [3,4,5], and *Aspergillus* spp., designated AFP and NFAP [6,7,8]. These proteins are of considerable interest for use as possible therapeutic agents against fungal diseases in humans, in food preservation, and in plant disease protection [9,10,11,12].

We are interested in a similar antifungal protein produced by *Epichloë festucae*, a fungal endophytic symbiont of the grass strong creeping red fescue (*Festuca rubra* subsp. *rubra*). Strong creeping red fescue is a commercially important low maintenance turfgrass species [13,14]. *Epichloë* spp. are endophytes of several cool-season grass species, often conferring insect resistance to their grass hosts due to the production of toxic alkaloids [15]. In *Epichloë*-infected forage grasses, the presence of some of the alkaloids can be toxic to grazing animals. Efforts to reduce or eliminate animal toxins while maintaining the insect toxins of the fungal endophytes is a topic of considerable research [16]. For turfgrasses, cultivars containing *Epichloë* endophytes are desired because of the enhanced insect resistance [17]. In addition to insect resistance, a unique feature of the strong creeping red fescue/*E. festucae* symbiosis is field level endophyte-mediated resistance to fungal pathogens [18,19]. Such endophyte-mediated disease resistance has not been reported in cultivated perennial ryegrasses (*Lolium perenne*) or tall fescues (*Lolium arundinaceum*) [19,20,21].

The mechanism underlying the unique endophyte-mediated disease resistance in strong creeping red fescue has not yet been established. We are pursuing the possibility that it may be due to the presence of an abundant secreted antifungal protein (gene model EfM3.063660; NCBI accession AWO72254) produced by the fungal endophyte *E. festucae*. The presence of the antifungal protein in *E. festucae* was first identified from a transcriptome study of endophyte-infected strong creeping red fescue [22]. The antifungal protein transcript was the second most abundant fungal transcript recovered in the study. The antifungal protein gene found in *E. festucae* infecting strong creeping red fescue is not present in most *Epichloë* spp. genomes for which whole genome sequences are available [20,22]. The transcript abundance and the limited existence of the gene among *Epichloë* spp. suggested the *E. festucae* antifungal protein may be a component of the unique endophyte-mediated disease resistance observed in strong creeping red fescue. Other antifungal compounds have been reported from some *Epichloë* spp. [16,23,24], but for commercially produced grasses, the field level disease resistance seen in *E. festucae*-infected fine fescues is unique.

The antifungal protein was expressed in the yeast *Pichia pastoris*, and the recombinant protein was shown to inhibit growth in culture of *Clarireedia jacksonii* (formerly *Sclerotinia homoeocarpa*), the causal agent of dollar spot disease [20,25]. *C. jacksonii* is a destructive fungal pathogen on many cultivated turfgrass species, particularly creeping bentgrass (*Agrostis stolonifera*), often requiring repeated applications of fungicides to suppress disease development [26,27]. Treatment of *C. jacksonii* mycelium with the antifungal protein resulted in uptake of the viability stains SYTOX Green and Evans blue, indicating that observed growth inhibition of the pathogen was due to damage to the plasma membrane of the pathogen [20]. These results support the hypothesis that the *E. festucae* antifungal protein is a component of the disease resistance seen in strong creeping red fescue.

The objective of this study was to determine if direct application of the *E. festucae* antifungal protein to susceptible turfgrass plants could protect them from injury caused by dollar spot pathogen. To do this, a simple method of protein purification was required. The yeast system was valuable for the initial demonstration that the purified *E. festucae* antifungal protein was active [20], but would be cumbersome for large-scale production of the protein. Unlike the similar proteins from *Penicillium* and *Aspergillus*, where their antifungal proteins could be easily purified from the culture filtrate, the *E. festucae* antifungal protein was not found in the culture filtrate, although it was highly expressed in the infected grass host [22]. Quantitative PCR analysis revealed the antifungal protein gene was expressed at a level greater than 700-fold higher in infected leaf sheath tissue than in culture [28]. Therefore, a simpler method of producing large quantities of the protein was sought. Here, we compare the activities and yields of the *E. festucae* antifungal protein expressed in *Escherichia coli*, *Pi. pastoris* and *Pe. chrysogenum,* and report the ability of the purified protein to reduce disease symptoms on plants of endophyte-free strong creeping red fescue and creeping bentgrass. We also report differences in the activity of *Efe*-AfpA with that of the similar protein PAF against *C. jacksonii* as well as wild type and glucosylceramide mutant strains of *Neurospora crassa.* Throughout this manuscript, we use the suggested nomenclature for *Epichloë* spp. genes and proteins [29]. The *E. festucae* antifungal protein gene is designated *Efe-afpA* and the protein is designated *Efe*-AfpA.

## 2. Materials and Methods

### 2.1. Primer Sequences

All oligonucleotide primer sequences used in this study are presented in Table S1.

### 2.2. Cloning of Modified E. festucae Antifungal Protein Coding Sequences in Escherichia coli

The mature *Efe*-*afpA* coding sequence [20] was modified and cloned into the pETite™ N-His SUMO vector according to the instructions of the Expresso T7 SUMO Cloning and Expression System (Lucigen Corp., Middleton, WI, USA). According to the system manual, large aliphatic residues following the SUMO tag, such as the N-terminal isoleucine of the mature *Efe*-AfpA, may result in slower cleavage rates by the SUMO protease. Both the mature *Pe. chrysogenum* antifungal protein (PAF) and the *A. giganteus* antifungal protein (AFP) start with an alanine. Therefore, the N-terminal isoleucine of *Efe*-AfpA cloned in the *Pi. pastoris* expression plasmid pPICZα A [20] was changed to alanine by using the Q5 Site-Directed Mutagenesis Kit (New England BioLabs Inc., Ipswich, MA, USA) according to the manufacturer’s instructions.

The N-terminal Ala-modified *Efe-afpA* coding sequence was then amplified by PCR with oligonucleotides that added sequences identical to the insertion site on the pETite™ N-His SUMO vector. The amplification reaction was carried out in a GeneAmp 9700 thermo-cycler (Applied Biosystems, Foster City, CA, USA). The 100 μL reactions contained 2X Phusion High-Fidelity PCR Master Mix with HF Buffer (Thermo Fisher Scientific, Waltham, MA, USA), 40 pmol of each oligonucleotide, and 25 ng plasmid DNA as template. The PCR reaction conditions were: an initial denaturation step at 98 °C for 30 s, followed by 30 cycles of 10 s denaturation at 98 °C, 30 s annealing at 59 °C, and 30 s extension at 72 °C. An additional final 5 min extension at 72 °C was performed.

After visualizing the PCR product on a 2% TBE agarose gel, 3 μL of the PCR product was mixed with 25 ng of pETite™ N-His SUMO vector and transformed directly into competent HI-Control 10G cells by heat shock in a 42 °C water bath for 45 s and returned to ice for 2 min. The transformed cells were incubated in Recovery Medium for 1 h at 37 °C with shaking, followed by overnight growth of cells on LB medium supplemented with 30 μg mL^−1^ kanamycin. Transformed bacterial colonies were screened for recombinant plasmids containing the *Efe-afpA* coding sequence inserts by using PCR with an initial denaturation step at 94 °C for 5 min, followed by 30 cycles of 30 s denaturation at 94 °C, 30 s annealing at 55 °C, and 30 s extension at 72 °C. An additional final 7 min extension at 72 °C was performed. The PCR products were visualized on a 2% TBE agarose gel. Plasmids from selected positive bacterial colonies were isolated and sequenced (Genewiz, Inc., South Plainfield, NJ, USA) using the SUMO Forward primer. One hundred ng of a plasmid containing the correct sequence was transformed into SHuffle T7 Express Competent *E. coli* (C3029, New England BioLabs Inc.) by heat shock in a 42 °C water bath for 30 s and returned to ice for 5 min. The transformed cells were incubated in SOC medium for 1 h at 30 °C with shaking, followed by 2 d growth of cells on LB medium supplemented with 30 μg mL^−1^ kanamycin.

Expression and purification of the N-terminal Ala modified *Efe*-AfpA (described below) revealed that the SUMO-protease reaction was not efficient in cleaving the SUMO tag with this modified version of *Efe*-AfpA. The Expresso T7 SUMO Cloning and Expression System (Lucigen Corp.) manual suggests that if incomplete digestion persists to insert or substitute Gly and or Ser residues next to the SUMO tag. Therefore, three additional N-terminal modifications were generated, (1) the N-terminal Ala was changed to Gly, (2) a Gly was added to the N-terminal Ala, and (3) Gly-Ser was added to the N-terminal Ala. The modifications were generated by using the Q5-Site Directed Mutagenesis Kit according to the manufacturer’s instructions. The resulting modified pETite™ N-His SUMO plasmids were then cloned into SHuffle T7 Express Competent *E. coli*.

### 2.3. Recombinant N-Terminal Modified Efe-AfpA Protein Purification

For purification of modified *Efe-*AfpA proteins, a starter culture of the Shuffle T7 cells containing the appropriate plasmid in 50 mL LB supplemented with 30 µg mL^−1^ kanamycin was grown overnight at 30 °C with shaking. The following day this was subcultured into 1 L LB plus 30 µg mL^−1^ kanamycin and shaken at room temperature until an OD_600_ of 0.6 to 0.8 was reached. *Efe*-AfpA expression was then induced by the addition of 4 mL of 100 mM IPTG with overnight shaking at room temperature until an OD_600_ of about 1 was reached. Recombinant *Efe*-AfpA proteins were purified by using TALON ^®^ Metal Affinity Resin (Takara Bio USA, Inc., San Jose, CA, USA). First, cells were collected by centrifugation followed by lysis using 100 mL 1× Fast Break Lysis Reagent (Promega Corporation, Madison, WI, USA) supplemented with 248 µL of 5 mg mL^−1^ DNase I (D4527, Sigma-Aldrich, St. Louis, MO, USA). The cells were rotated for 20 min to allow for complete lysis. The 6xHIS-SUMO tagged *Efe*-AfpA proteins were isolated by the addition of pre-equilibrated TALON resin and incubated for 20 min with rotation. The resin was collected by centrifugation and the supernatant decanted. The resin was then washed in TALON equilibration buffer, applied to a column, and bound SUMO-tagged *Efe*-AfpA was eluted using TALON Elution Buffer. High-molecular-weight proteins were removed from the eluate by centrifugation through a 30 kDa Amicon ^®^ Ultra-15 Centrifugal Filter Unit (MilliporeSigma, Burlington, MA, USA). The protein in the flow-through was concentrated and the elution buffer was exchanged to 50 mM NaPO_4_, pH 7.0, 300 mM NaCl by using a 3 kDa Amicon ^®^ Ultra-15 Centrifugal Filter Unit. To remove the 6xHis-SUMO tag from the modified forms of *Efe*-AfpA, 100 µg of purified 6xHis-SUMO-tagged protein was incubated overnight at 4 °C with 1 unit of SUMO protease (Lucigen Corp.). The buffer of the digested *Efe*-AfpA solution was exchanged to 50 mM NaPO_4_, pH 7.0, to reduce the salt concentration, by using a 3 kDa Amicon ^®^ Ultra-15 Centrifugal Filter Unit. The released *Efe*-AfpA protein was then separated from the 6xHis-SUMO tag by batch ionic exchange purification by using carboxymethyl cellulose (CMC52) (Biophoretics, Sparks, NV, USA). CMC52 was pre-equilibrated with 50 mM NaPO_4_ pH 7.0 for 1 h, added to the digest solution, and incubated for 3 h at 25 °C. At pH 7.0, *Efe*-AfpA (pI = 8.9) is positively charged and binds to the CMC52. The CMC52 was applied to a column, washed with excess 50 mM NaPO_4_, pH 7.0, and *Efe*-AfpA was eluted with NaCl amended buffer ranging from 0.1 to 0.5 M. The A280 of the eluted fractions was monitored by using a NanoDrop spectrophotometer (Thermo Fisher Scientific). Protein concentrations of the fractions were determined by using the molecular weight of *Efe*-AfpA (6.278 kDa) and the predicted extinction coefficient of 5220 M^−1^ cm^−1^ [30]. Protein containing fractions were then concentrated and the buffer exchanged to sterile water by using a 3 kDa Amicon ^®^ Ultra-15 Centrifugal Filter. The purified *Efe*-AfpA solution was sterilized by filtering the protein through a 0.2 µm polyethersulfone syringe filter (Corning Inc., Corning, NY, USA).

### 2.4. Purification of Efe-AfpA from Culture Filtrates of Pichia pastoris

The cloning of *Efe*-AfpA in *Pi. pastoris* was described previously [20]. Expression and purification of *Efe*-AfpA is as described below. *Pi. pastoris^Efe^*^AfpA^ was streaked onto solid YPD (1% yeast extract, 2% peptone, 2% dextrose, 2% agar) plates amended with 100 µg mL^−1^ Zeocin, and incubated at 30 °C until single colonies appeared. A single colony was grown in a starter culture of 50 mL BMGY (1% yeast extract, 2% peptone, 100 mM potassium phosphate pH 6.0, 1.34% yeast nitrogen base, 4 × 10^−5^% biotin, 1% glycerol) at 30 °C with shaking at 200 rpm until an OD_600_ of at least 2 was reached. The culture was then subcultured into 1 L of fresh BMGY and grown at 30 °C with shaking at 200 rpm until the OD_600_ was at least 2. The cells were then pelleted by centrifugation and resuspended in 1 L BMMY (1% yeast extract, 2% peptone, 100 mM potassium phosphate pH 6.0, 1.34% yeast nitrogen base, 4 × 10^−5^% biotin, 0.5% methanol) to induce expression of the *Efe*-AfpA protein. Expression of *Efe*-AfpA was induced with the addition of 5 mL of methanol daily for 5 days. The cells were then pelleted by centrifugation at 10,000 rpm for 10 min, and the culture supernatant transferred to SnakeSkin^TM^ Dialysis Tubing (Thermo Fisher Scientific) with a molecular weight cut-off of 3.5 K and was dialyzed overnight in 8 L of 10 mM NaPO_4_ pH 6.6, 25 mM NaCl, 0.15 mM EDTA. Dialyzed culture supernatant was then applied to a CMC52 column pre-equilibrated in 10 mM NaPO_4_, pH 6.6, 25 mM NaCl, 0.15 mM EDTA buffer. The column was washed with excess buffer and eluted with increasing salt concentrations from 0.1 to 0.5 M NaCl. Protein concentration in the fractions was determined as described above and the protein containing fractions were then filtered through a 30 kDa Amicon ^®^ Ultra-15 Centrifugal Filter to remove high molecular weight proteins. The flow-through containing *Efe-*AfpA was then concentrated and the buffer exchanged to sterile water by using a 3 kDa Amicon ^®^ Ultra-15 Centrifugal Filter. The purified *Efe*-AfpA was then sterilized by filtering the protein through a 0.2 µm polyethersulfone syringe filter.

### 2.5. Cloning and Transformation of Efe-afpA into Penicillium chrysogenum

The pSK275*paf* plasmid and a *Pe. chrysogenum* isolate in which the *paf* gene was deleted were provided by Dr. Florentine Marx. The pSK275*paf* plasmid was developed for expressing the *Pe. chrysogenum* antifungal protein *paf* and *paf* variants in the *Pe. chrysogenum* strain in which the *paf* gene had been deleted, and includes the *paf* promoter and coding sequences [31]. The pSK275*paf* plasmid was modified by cloning the *Efe-afpA* coding sequence of the mature protein in place of the *paf* coding sequence of the mature protein. This generated a plasmid in which the *Efe-afpA* coding sequence of the mature protein was downstream of the *Pe. chrysogenum paf* promoter and *paf* signal peptide and propeptide.

Assembly of the pSK275:*Efe*-*AfpA* vector was completed using the NEBuilder^®^ HiFi DNA Assembly Kit (New England Biolabs Inc.) The mature *Efe*-AfpA sequence was produced by PCR from the pETite:*Efe*-*afpA* vector (described above) with overhangs homologous to regions on the pSK275*paf* plasmid. The pSK275*paf* backbone fragments were also generated by PCR, and the final pSK275:*Efe*-*afpA* vector was constructed following the HiFi DNA Assembly Kit manual. Transformation of the *Pe. chrysogenum* Δ*paf* strain was completed as described previously [31]. Spores (2 × 10^8^) of the Δ*paf* strain were inoculated in 200 mL complete medium [31] and shaken at 200 rpm at room temperature for 48 h. Fungal pellets were harvested by filtering through cheesecloth and converted to protoplasts by using 1.2 g Vinotaste Pro (Novozymes, Franklinton, NC, USA) in 30 mL lytic buffer (50 mM potassium phosphate, 0.7 M KCl, pH 5.8). Protoplasts were harvested by filtration through filter paper (Whatman Grade 1) and then pelleted by centrifugation (3000 rpm). The protoplasts were then washed twice with 0.7 M KCl, resuspended in a final volume of 300 µL 0.7 M KCl, and counted. Ten to fifteen µg of pSK275:*Efe*-*afpA*, linearized by digestion with Not1, were added to 100 µL of protoplasts (10^8^) followed by 25 µL PCM (0.8% CaCl_2_, 2% MOPS, 50% PEG 6000, pH 5.8). Both the transformation solution and appropriate control solutions were incubated on ice for 30 min. PCM (250 µL) was then added to both the sample and controls, and they were further incubated for 20 min at room temperature. Lastly, 1 mL of 0.7 M KCl was added and both transformation and control solutions were mixed in melted PcMM (*Pe. chrysogenum* Minimal Media, 0.3% NaNO_3_, 0.05% MgSO_4_ × 7H_2_O, 0.05% KCl, 0.005% FeSO_4_ × 7H_2_O, 2% sucrose, 2.5% 1M potassium phosphate buffer pH 5.8, 0.1% trace elements solution A) top agar (1% agar) [31] prior to being plated on antibiotic amended (0.6 µg mL^−1^ pyrithiamine and 200 µg mL^−1^ noureseothricin) PcMM bottom agar (2% agar). Trace elements solution A was 0.1% FeSO_4_•7H_2_O, 0.9% ZnSO_4_, 0.04% CuSO_4_, 0.01% MnSO_4_, 0.01% H_3_BO_3_, 0.01% Na_2_MoO_4_.

Plates were incubated at 25 °C for up to two weeks to allow transformants to grow. Colonies were then subcultured onto fresh PcMM_Pyr0.6Nour200_ and screened for gene integration by spore PCR using the primers *Efe*-AfpA Forward and *Efe*-AfpA Reverse with positive transformants yielding a band on a 2% agarose gel of 168 bp. Positive colonies were then subjected to single spore selection, allowing for the collection of a genetically homogeneous strain. PCR positive transformants from single spore isolates were then streaked onto PcMM_Pyr0.6Nour200_. Spores were collected and inoculated into 25 mL liquid PcMM. After seven days of growth at room temperature with shaking at 200 rpm, the culture supernatant was collected, filtered through a 30 kDa Amicon ^®^ Ultra-15 Centrifugal Filter Unit and concentrated on a 10 kDa Amicon ^®^ Ultra-15 Centrifugal Filter Unit (Millipore). Supernatants were then quantitated by A_280_ (Nanodrop ND-1000, Thermo Fisher Scientific) and equal total protein amounts were compared by 16% SDS PAGE to visualize the secreted *Efe*-AfpA protein. Transformants with the highest levels of *Efe*-AfpA were chosen for further analysis. Primers pSK275:*Efe*-AfpA F and pSK275:*Efe*-AfpA R were used to amplify the *Efe*-AfpA gene region of the chosen transformants. The PCR fragment was gel purified using the QIAquick Gel Extraction Kit (Qiagen Inc, Germantown, MD, USA) and sequenced (Genewiz Inc., South Plainfield, NJ, USA).

### 2.6. Purification of Efe-AfpA from Culture Filtrates of Penicillium chrysogenum and of PAF from an Overexpression Strain of Pe. chrysogenum

A strain of *Pe. chrysogenum* that overexpressed PAF (designated *Pe. chrysogenum paf*) [31] was used to purify PAF for comparison of activity with *Efe*-AfpA purified from the different expression systems. Purification of PAF and *Efe*-AfpA from *Pe. chrysogenum* was as described below.

*Pe. chrysogenumpaf* conidia were streaked onto solid PcMM 2% agar plates supplemented with 200 µg mL^−1^ nourseothricin and 0.6 µg mL^−1^ pyrithiamine from freezer stocks and grown for 4 days at room temperature. Spores were then harvested in spore buffer (0.9% NaCl, 0.01% Tween 20), washed twice in spore buffer, and counted using a hemocytometer. Conidia (2 × 10^8^) were inoculated into 200 mL of PcMM and grown at room temperature with shaking at 200 rpm for 72 h. The culture supernatant was filtered through cheesecloth to remove mycelia and any excess debris was pelleted by centrifugation at 10,000 rpm for 10 min. The culture supernatant was then applied to a CMC52 column pre-equilibrated in 10 mM NaPO_4_, 25 mM NaCl, 0.15 mM EDTA, pH 6.6 buffer. The column was washed with excess buffer and eluted with increasing salt concentrations from 0.1 to 0.5 M NaCl. Eluted fractions were then evaluated for the presence of protein at A_280_ (Nanodrop ND-1000) utilizing the molecular weight (6.25 kDa) and extinction coefficient (4845 M^−1^ cm^−1^) of PAF [32]. The protein-containing fractions were then filtered through a 30 kDa Amicon ^®^ Ultra-15 Centrifugal Filter, concentrated and desalted using a 3 kDa Amicon ^®^ Ultra-15 Centrifugal Filter and sterile distilled water, and sterilized by filtering the protein through a 0.2 µm polyethersulfone syringe filter.

*Pe. chrysogenum^Efe^*^-AfpA^ required an altered cultivation method for protein expression due to the low production of *Efe*-AfpA when using the method described above for PAF production. This was likely due to *Efe*-AfpA activity against *Pe. chrysogenum*, as discussed below. *Pe. chrysogenum^Efe^*^-AfpA^ conidia from freezer stocks were streaked onto solid PcMM 2% agar plates supplemented with 200 µg mL^−1^ nourseothricin and 0.6 µg mL^−1^ pyrithiamine and grown for 4 days at room temperature. Spores were then harvested in spore buffer, washed twice in spore buffer, and counted using a hemocytometer. Conidia (2 × 10^8^) were inoculated into 200 mL of *A. nidulans* complete media [31] and grown at room temperature for 48 h with shaking at 200 rpm. Mycelia were harvested on cheesecloth, washed with sterile distilled water, and subcultured into 200 mL PcMM to induce expression of the *Efe*-AfpA protein for 72 h at room temperature with shaking at 200 rpm. Purification of *Efe*-AfpA from the culture filtrate was as described above for purification of PAF. For *Efe*-AfpA quantitation, the molecular weight 6.278 kDa and the theoretical extinction coefficient 5220 M^−1^ cm^−1^ were used [30].

### 2.7. Neurospora crassa Conidial Growth Assays

The antifungal activities of the purified forms of N-terminal modified *Efe*-AfpA expressed in *E. coli* were compared in a *Neurospora crassa* (wild type strain 74-OR23-IVA; Fungal Genetics Stock Center #2489) conidial growth assay carried out in 96-well microtiter plates. The assay was based on the method described previously [33]. Frozen *N. crassa* conidia were thawed and 50 μL were diluted in 1 mL of 2× low-cationic medium (LCM) (1× LCM is 2 g L^−1^ glucose, 0.1 g L^−1^ yeast extract, 0.05 g L^−1^ peptone). The sample was counted by using a hemocytometer and further diluted with 2X LCM to a final working concentration of 2 × 10^6^ conidia mL^−1^. Solutions (100 µL) of varying amounts of the purified antifungal proteins in 20 mM Tris-Cl, pH 8.0, 150 mM NaCl buffer were mixed with 100 uL of 2 × 10^6^ *N. crassa* conida for a final volume of 200 uL in each well. Each sample was assayed in triplicate. The untreated control sample was 100 μL conidia + 100 μL 20 mM Tris, pH 8.0, 150 mM NaCl. The optical density at 620 nm was measured immediately with a microtiter plate reader (Absorbance 96, Byonoy GmbH, Hamburg, Germany) and the plates incubated at room temperature. The optical density was measured again after 24 h incubation and the initial readings subtracted from the 24 h readings. The corrected optical density of the untreated control conidia was considered 100% growth (0% inhibition). The percentage inhibition of the *Efe*-AfpA treated samples was calculated by comparing the conidia growth in the absence of *Efe*-AfpA to the growth in the treated samples.

The antifungal activities of the purified *Efe*-AfpA proteins expressed in *Pi. pastoris* and *Pe. chrysogenum* and of PAF from *Pe. chrysogenum paf* were compared in a similar *N. crassa* wild type conidial growth assay carried out in 96-well microtiter plates.

### 2.8. Penicillium chrysogenum Δpaf Sensitivity to Efe-AfpA

The recipient strain *P. chrysogenum* Δ*paf* was cultured as described above on solid antibiotic amended PcMM. Spores were harvested in spore buffer, washed twice with excess spore buffer, washed once in 2× LCM, and resuspended in fresh 2× LCM. Spores were counted with a hemocytometer and diluted to a working concentration of 2 × 10^4^ conidia mL^−1^. One hundred µL of spores were mixed with 100 µL of 10 µg mL^−1^ *Efe*-AfpA or PAF resulting in a final concentration of 1 × 10^4^ conidia mL^−1^ spores and 5 µg mL^−1^ protein in a 96-well plate in triplicate. Sterile distilled water was used as the control. Growth was monitored by absorbance at 620 nm in a microtiter plate reader (Absorbance 96, Byonoy GmbH). Absorbances at 30 h were corrected by subtracting the initial absorbances at 0 h. The water treated wells were considered 100% growth (0% inhibition). Wells were also visualized microscopically (EVOS M5000, Invitrogen).

### 2.9. Clarireedia jacksonii Inhibition Assays

A *C. jacksonii* isolate recovered from the creeping bentgrass cultivar ‘Luminary’ was maintained on 1× potato dextrose agar (PDA). Since *C. jacksonii* does not produce spores, it was maintained by routinely transferring plugs from the growing edge of a plate to a new PDA plate.

The Evans blue staining assay was modified from that previously described [20,34]. One mL PDA was applied to a surface sterilized microscope slide making a 3 cm by 2.5 cm rectangle of PDA. A 5 mm plug of *C. jacksonii* was taken from a 4-day old PDA plate and placed on the left edge of the PDA rectangle on the slide. The microscope slides were incubated at room temperature for 24 h in a closed Petri dish. The next day, 10 µL containing 300 ng of *Efe*-AfpA or PAF, or water were placed at the growing edge of the *C. jacksonii* mycelium. The microscope slides were then incubated for another 24 h at room temperature to allow the mycelium to grow into the treatment. The mycelium was then stained in 1% Evan’s blue stain for 15 min and destained in distilled water. Mycelium was then visualized microscopically. Each treatment had 3 replicates, and the experiment was completed twice.

For plate assays, 35 mm Petri dish plates containing 8 mL of PDA amended with increasing concentrations of *Efe*-AfpA, PAF, or water were used. Concentrations of the antifungal proteins tested were 0, 0.5, 1, 10, 20, 30, 40, 50, 100 µg mL^−1^. A 5 mm plug of *C. jacksonii* was taken from a 4-day-old PDA plate and placed in the center of each 35 mm plate. Plates were incubated at room temperature for 4 days. Plates were photographed, and cross section mycelium diameters were measured daily. Each treatment had three replicates and the experiment was completed twice.

### 2.10. Strong Creeping Red Fescue and Creeping Bentgrass Greenhouse Infection Assay

Strong creeping red fescue and creeping bentgrass plants were maintained in a greenhouse in Pro-Mix BX potting mix (Premier Horticulture Inc., Quakertown, PA, USA).

The strong creeping red fescue plants used for the greenhouse assays were an endophyte-free line S1139E and the same genotype infected with the *E. festucae* Rose City isolate, S1139RC. The generation of these plants was described previously [35]. Plants were repotted into 14.6 cm diameter pots prior to infection and trimmed to even heights. Pots of similar density were chosen and inoculated with either an 8 mm plug of *C. jacksonii* grown for 5 days on PDA or an 8 mm plug of PDA. To ensure the high humidity required for infection, the plants were covered with a translucent plastic bag, and placed in a tray containing a shallow layer of water. The plants were sprayed daily for 10 days with either 1 mL of water containing 0.1% Tween 20 or 1 mL 100 µg mL^−1^ *Efe*-AfpA containing 0.1% Tween 20. Four replicates were used per treatment. Photographs were taken ten days post inoculation.

Creeping bentgrass ‘Crenshaw’ plugs (10 cm diameter × 6 cm depth) were taken from the Rutgers University Horticulture Farm 2 experimental research plots in North Brunswick, New Jersey, transferred to 10 cm × 10 cm pots, and maintained in the greenhouse. The plants were propagated by repotting as needed. For the disease assays, plants were repotted to 8 × 8 cm pots. Prior to inoculation, creeping bentgrass plants of similar density were chosen and trimmed to the same height. For the fungal inoculum, 8 mm plugs were harvested from the growing edge of 5-day-old *C. jacksonii* cultures on PDA plates, as well as 8 mm plugs of PDA for the controls. A plug of either *C. jacksonii* or PDA was placed in the center of a pot resting on the grass blades, the plants were covered with a translucent plastic bag, and placed in a tray containing a shallow layer of water. Each pot was then sprayed daily with either 1 mL of water containing 0.1% Tween 20 or 1 mL of *Efe*-AfpA at various concentrations with 0.1% Tween 20. Six replicates were used per treatment. Photographs were taken 7 days post inoculation.

The timing of applications was also tested. Pots of creeping bentgrass plants and *C. jacksonii* inoculum were prepared as described above. Either 1 mL of water containing 0.1% Tween 20 or 1 mL of 100 µg mL^−1^ *Efe*-AfpA containing 0.1% Tween 20 was sprayed onto the pots at 0 h, 0 and 48 h, or daily for a total of one, two, or seven applications, respectively. Six replicates were used per treatment. Photographs were taken 7 days post inoculation.

### 2.11. Evaluation of the Effect of Neurospora crassa Glucosylceramides on Efe-AfpA Activity

To compare the activity of *Efe*-AfpA to PAF and evaluate the effect of *N. crasssa* glucosylceramides on *Efe*-AfpA activity, the assay was carried out on conidia of the glucosylceramide synthase [36] and 2-hydroxy fatty N-acyl-Δ3(E)-desaturase [37] deletion mutants, Fungal Genetics Stock Center #13794 and FGSC #22453, respectively [38,39].

*N. crassa* wild type, Δ*gcs,* and Δ*dtd* strains were grown on Vogel’s solid media (supplemented with 200 µg mL^−1^ hygromycin for the two knockout strains). The three strains were streaked onto fresh Vogel’s media from freezer stocks and incubated at room temperature for four to five days to allow for spore generation. Spores were harvested and washed as described above, diluted in 2× LCM to a working concentration of 2 × 10^4^ conidia mL^−1^. One hundred µL of spores were incubated with 100 µL of water or increasing concentrations of either *Efe*-AfpA or PAF to final concentrations of 0.3, 0.6, 1.2, 5, 10, 20, 30, 40, 50, and 100 µg mL^−1^. Growth was monitored by absorbance at 620 nm at 0 and 24 h as described above. Untreated spores were considered 100% growth as compared to treated spores. All treatments were done in triplicate and the experiments were completed at least three times. Minimal inhibitory concentration (MIC) was determined from the absorbance data. MIC is considered the concentration of protein required for ≥ 90% inhibition of growth. Samples were also visualized microscopically (EVOS M5000, Invitrogen).

To further determine the susceptibility of the three *N. crassa* strains to *Efe*-AfpA, spores of each were cultured on solid Vogel’s media supplemented with increasing concentrations of either *Efe*-AfpA or PAF (0–100 µg mL^−1^) in 24-well plates [36]. Five µL of each strain at a concentration of 2 × 10^5^ conidia mL^−1^ were plated on 500 µL Vogel’s media and incubated at room temperate for 96 h and photographed at 72 h. The experiment was done in duplicate per protein and completed twice.

*Efe*-AfpA’s effect on conidia germination of the three *N. crassa* strains was determined by modifying the method previously described [40]. One hundred µL of *N. crassa* conidia (1 × 10^5^ mL^−1^) were incubated with 100 µL of 20 µg mL^−1^ *Efe*-AfpA or PAF, or water for 6 h in a 96 well plate at room temperature. Samples were also visualized microscopically, and 100 conidia were counted from each replicate per treatment. Germination efficiency was calculated as the percent of the 100 conidia that germinated. Each treatment was done in triplicate and the experiment was completed three times.

### 2.12. Phylogenetic Analysis

The Clustal-X program [41] was used to align the full antifungal protein amino acid sequences, including the signal peptides. The phylogenetic analysis was performed with the Paup* program, version 4.0b10 for Macintosh. The phylogenetic analysis was done by using the maximum parsimony full heuristic search option set to random sequence addition, tree-bisection-reconnection (TBR) branch swapping, and Multrees on, with 1000 bootstrap replications. Gaps were treated as missing.

### 2.13. Protein Gel Electrophoresis

For sodium dodecyl sulfate-polyacrylamide gel electrophoresis (SDS-PAGE) analysis, protein samples were mixed with 5× SDS sample buffer (5:1, *v*/*v*), [42], then boiled for 5 min and subjected to electrophoresis in 16% polyacrylamide gels. Gels were stained with Coomassie Blue to visualize protein bands. Gels were destained and then dried as described previously [43].

## 3. Results

### 3.1. Production of Efe-AfpA in E. coli

Bacterial expression of mature *Efe*-AfpA was achieved from a plasmid that adds a cleavable N-terminal 6xHis-SUMO (small ubiquitin-like modifier) tag [44] in the engineered *E. coli* strain SHuffle, which maintains an oxidative cytoplasmic environment allowing disulfide bond formation in the recombinant protein [45]. Modification of the mature N-terminus of *Efe*-AfpA was required since the native N-terminal isoleucine is known to inhibit cleavage of the SUMO tag by the SUMO protease. Four modified N-terminal coding sequences of the mature form of *Efe*-AfpA were cloned into the *E. coli* expression vector pETite™ N-His-SUMO and the resulting plasmids cloned into SHuffle competent cells. The modifications and the resulting protein designations are presented in Table 1.

In all cases, the 6xHis-SUMO-tagged *Efe*-AfpA was a major soluble protein after induction by IPTG and could be purified by binding to TALON resin. The 6xHis-SUMO tag could be cleaved by digestion with an engineered 6xHis-tagged SUMO protease, releasing *Efe*-AfpA from the SUMO tag. Efe-AfpA was separated from the released tag and the SUMO protease by binding to CMC52. Three of the four N-terminal modified *Efe*-AfpA proteins were efficiently cleaved by the SUMO-protease (Figure S1). The A-*Efe*-AfpA-modified protein was not efficiently cleaved by the protease, even on extended incubation, and was therefore dropped from further characterization.

### 3.2. Activity of the Modified Efe-AfpA Proteins

**Figure 1:**
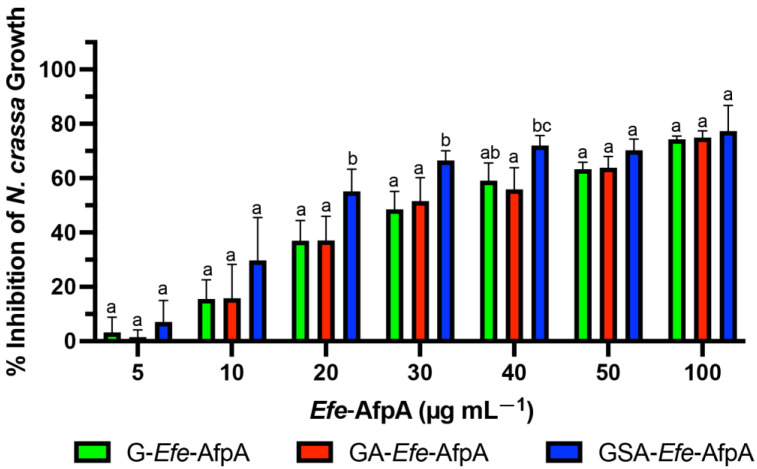
Comparison of activity of bacterially produced *Efe*-AfpA with varying N-terminal amino acids. Increasing concentrations of the modified *Efe*-AfpA proteins were assayed for activity in the *N. crassa* growth assay with 1 × 10^6^ conidia mL ^−1^. The data presented are the means and standard deviations of three replicates. For each concentration, columns with different letters indicate a significant difference in activity (*p* ≤ 0.05, two-way ANOVA).

The antifungal activity of the N-terminal-modified *Efe*-AfpA proteins produced in *E. coli* was quantitatively assessed against *N. crassa* conidia germination in a microtiter plate assay. *N. crassa* has been established as a sensitive model system for testing the antifungal activity of the similar *Pe. chrysogenum* protein, PAF [46]. All three of the tested N-terminal modified forms of *Efe*-AfpA had activity at all the concentrations tested, with the GSA-*Efe*-AfpA form having statistically significant higher inhibition at 20, 30 and 40 µg mL^−1^ (Figure 1). At 100 µg mL^−1^, the inhibition of *N. crassa* growth was similar with all modified forms, ranging from 74 to 77%.

### 3.3. Expression of Efe-AfpA in Penicillium chrysogenum

**Figure 2:**
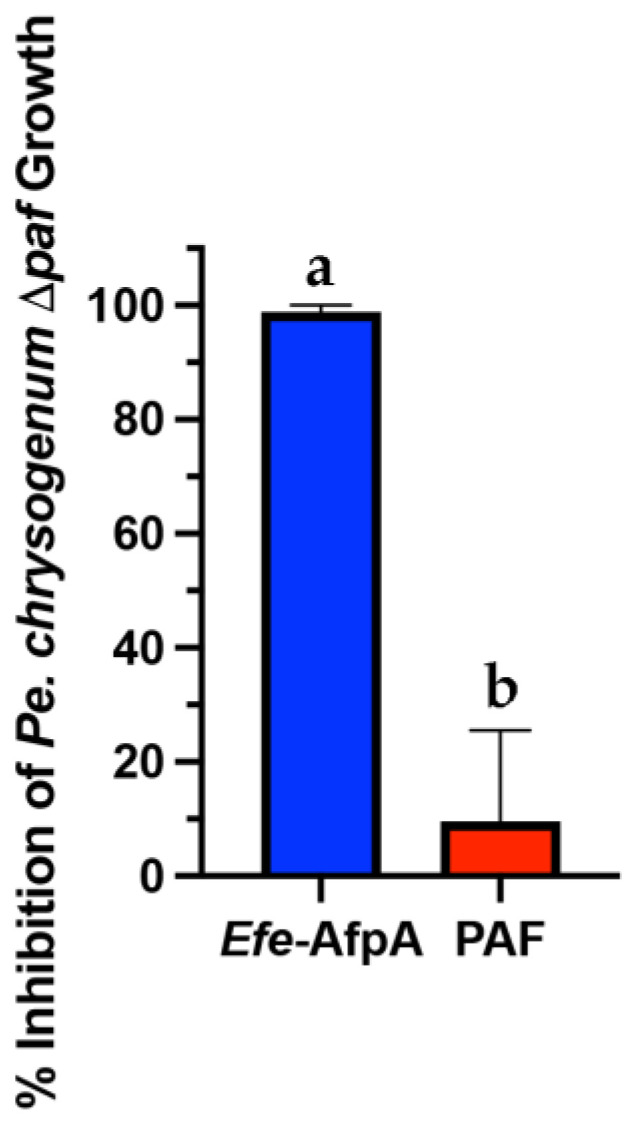
Comparison of activity of *Efe*-AfpA and PAF against *Pe*. *chrysogenum* Δ*paf* conidial growth. Five µg mL^−1^ of either *Efe*-AfpA or PAF was assayed for activity against 1 × 10^4^ conidia mL^−1^. The data presented are the means and standard deviations of three replicates. Columns with different letters indicate a significant difference in activity (*p* ≤ 0.05, one-way ANOVA).

*Pe. Chrysogenum*, the producer of the antifungal protein PAF, has been developed as an efficient expression system for other antifungal proteins [31]. In this system the expression of the target protein is driven by the native *paf* promoter in a strain in which the *paf* gene has been deleted. Although active *Efe*-AfpA could be recovered from the *Pe. Chrysogenum* expression system when using the growth conditions used for PAF [31], yields were variable and in some cases the *Pe. Chrysogenum* cells appeared to lyse suggesting that *Efe*-AfpA may have activity against *Pe. Chrysogenum*. To test this possibility, the activity of *Efe*-AfpA on *Pe. Chrysogenum* conidial growth was assayed as for *N. crassa*. *Efe*-AfpA nearly completely inhibited *Pe. Chrysogenum* conidial growth even at the low concentration of 5 µg mL^−1^, whereas PAF was not inhibitory (Figure 2).

**Figure 3:**
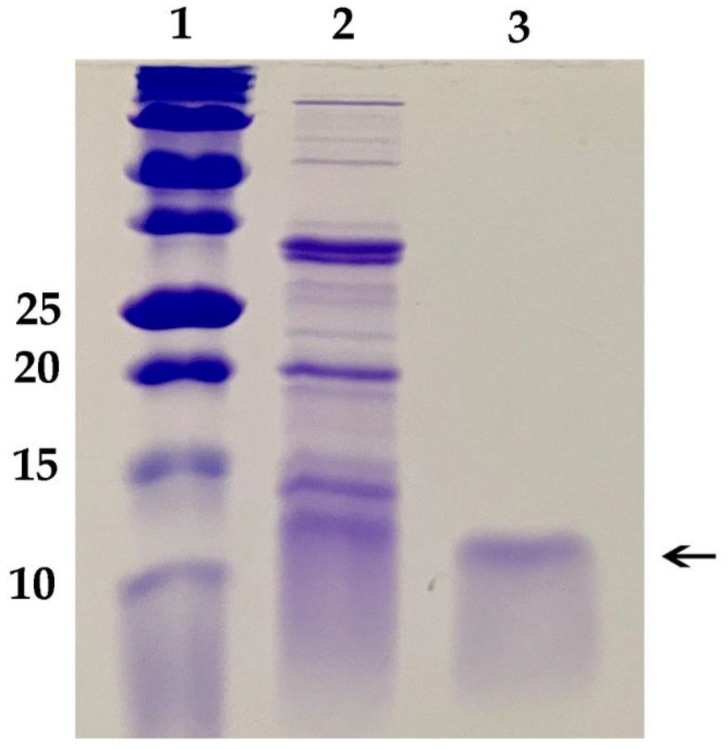
SDS polyacrylamide gel of purification of the *E. festucae* antifungal protein produced in *Pe. chrysogenum*. Lane 1, Bio-Rad Precision Plus Protein Dual Xtra Standards, size of markers in kD given on left; Lane 2, Crude culture filtrate of *Pe. chrysogenum* expressing the antifungal protein; Lane 3, 1 µg purified *Efe*-AfpA, indicated by the arrow.

These results necessitated development of a new protocol for growth of *P. chrysogenum* expressing *Efe*-AfpA to avoid the toxic effects of the *Efe*-AfpA on *Pe. chrysogenum*. A successful protocol was developed that paired growth of the fungus for 48 h in a high nutrient medium that did not induce expression of *Efe*-AfpA, followed by transfer of the fungal mycelium to a low nutrient medium for 48 to 72 h. In the high nutrient medium, the fungus grew rapidly generating a large biomass of mycelium. In the low nutrient medium *Efe-*AfpA was expressed and secreted to the surrounding medium from the large biomass, resulting in high yields of active protein*. Efe*-AfpA was purified from the culture medium using a combination of cation exchange and size exclusion filtration (Figure 3). Although the calculated size of the mature protein is 6278 Daltons, it ran on a gel at a position similar to the 10 kDa marker.

**Figure 4:**
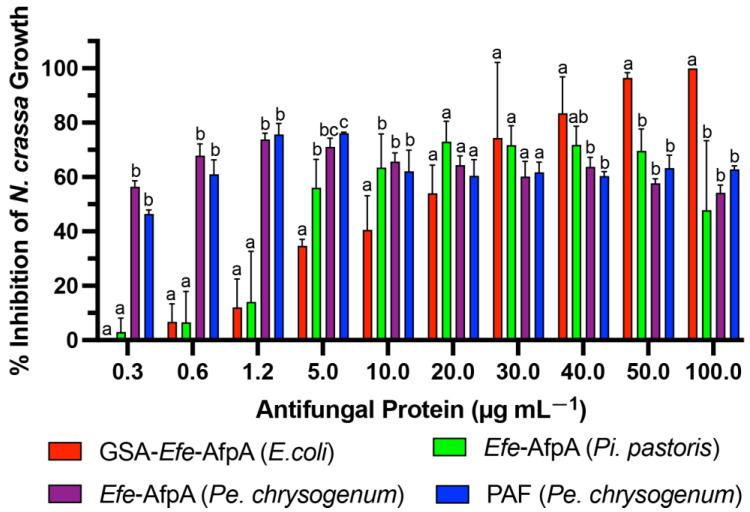
Comparison of activities of PAF purified from *Pe. chrysogenum* and *Efe*-AfpA purified from *E. coli*, *Pi. pastoris*, and *Pe. chrysogenum*. Increasing concentrations of the antifungal proteins were assayed for activity in the *N. crassa* growth assay with 1 × 10^6^ conidia mL^−1^. The data presented are the means and standard deviations of three replicates. For each concentration, columns with different letters indicate a significant difference in activity (*p* ≤ 0.05, two-way ANOVA).

*Efe*-AfpA was expressed in the *Pe. chrysogenum* system and the activity of the purified protein was compared with the activity when expressed in *Pi. pastoris* and in *E. coli*, as well as with PAF, in the *N. crassa* conidial growth assay (Figure 4). The activity of *Efe*-AfpA expressed in *Pe. chrysogenum* was similar to the activity of PAF at all concentrations tested. At lower concentrations, *Efe*-AfpA expressed in *Pe. chrysogenum* had significantly higher activity than when produced in *Pi. pastoris* and *E. coli*. At 20 and 30 µg mL^−1^ the activity of *Efe*-AfpA expressed in all three systems was similar. At the higher concentrations, 40, 50, and 100 µg mL^−1^, *Efe*-AfpA produced in *E. coli* had significantly higher activity than the protein expressed in *Pi. pastoris* or *Pe. chrysogenum*. However, the significantly lower yields of pure protein from the *E. coli* system makes that system impractical for large-scale production of the protein (Table 2). The *Pe. chrysogenum* system was therefore chosen as the optimal system for further characterization of *Efe*-AfpA since it produced the highest quantity of *Efe*-AfpA and was the most convenient for purification. The difference in activity in *Efe*-AfpA produced in the different expression systems may be due to a difference in proper folding of the protein, which is known to affect the activity of the similar protein PAF [33].

### 3.4. Activity of Efe-AfpA and PAF against Clarireedia jacksonii in Culture

**Figure 5:**
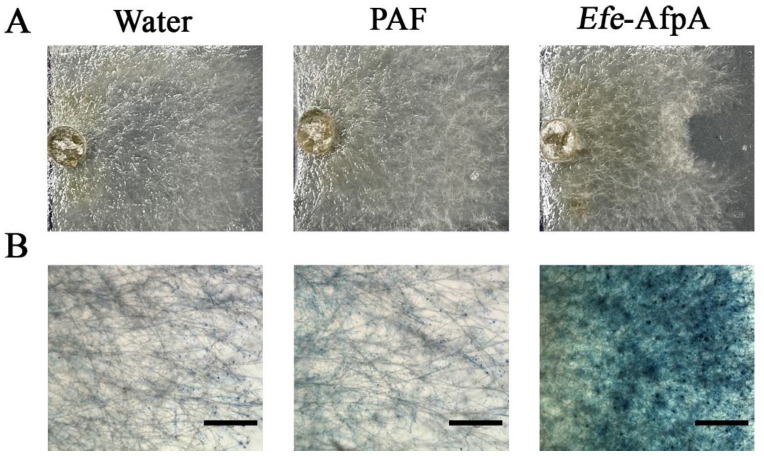
Comparison of the activity of *Efe*-AfpA expressed in *Pe. chrysogenum* with PAF, a similar antifungal protein from *Pe. chrysogenum*, against *C. jacksonii* in culture. In the upper panels (**A**) water (10 µL), PAF (300 ng), or purified *Efe-*AfpA (300 ng), was placed on the right side of a plug of *C. jacksonii*. The lower panels (**B**) show the *C. jacksonii* hyphae from the upper panels treated with Evans blue. Bars in the lower panels are 750 μm.

Previously, *Efe*-AfpA produced in *Pi. pastoris* was reported to have activity in culture against *C. jacksonii*, the dollar spot pathogen of turfgrasses [20]. Since the activity of *Efe*-AfpA produced in *Pe. chrysogenum* was similar to that of PAF in the *N. crassa* assay, the activity of the two antifungal proteins against *C. jacksonii* was compared in culture (Figure 5). Efe-AfpA clearly inhibited the growth of the *C. jacksonii* mycelial plug, whereas PAF did not (Figure 5A). The dye Evans blue enters cells that have damaged cell membranes [47]. Treatment of *C. jacksonii* with the *E. festucae* antifungal protein resulted in damage to the cell membranes as evidenced by Evans blue staining, whereas treatment with PAF did not (Figure 5B).

**Figure 6:**
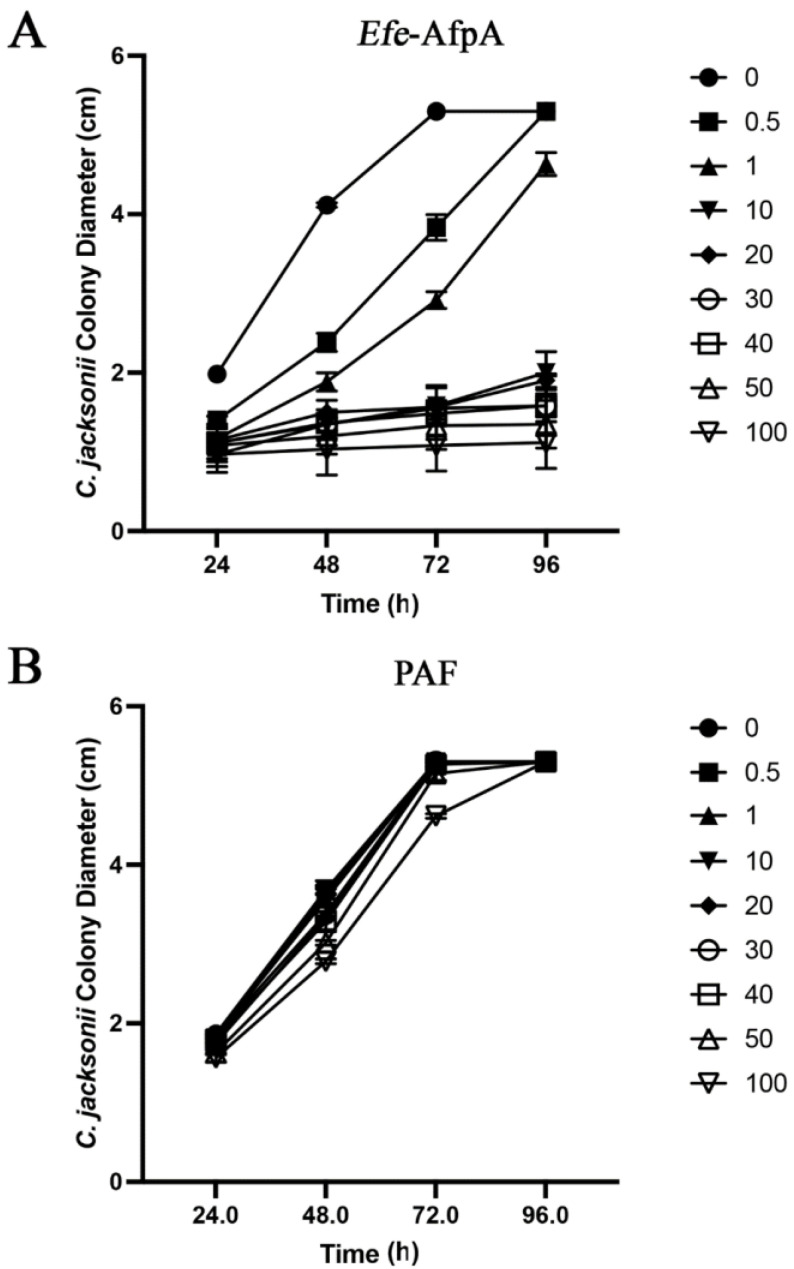
Effect of *Efe*-AfpA (**A**) or PAF (**B**) on *C. jacksonii* mycelial growth. *C. jacksonii* mycelial plugs were subcultured onto PDA plates amended with increasing concentrations of either *Efe*-AfpA or PAF. The colony diameters were measured daily. The data presented are the means and standard deviations of three replicates. Photographs of the plates are shown in Figures S2 and S3.

In agar plate assays in which either purified *Efe-*AfpA or PAF were incorporated into the agar, *Efe*-AfpA inhibited *C. jacksonii* growth at concentrations of 0.5 μg mL^−1^ and higher, whereas PAF was not inhibitory at any concentration (Figure 6, Figure S1 and Figure S2). Although PAF is similar to *Efe*-AfpA in amino acid sequence and in activity against *N. crassa*, it did not have activity against *C. jacksonii* in culture.

### 3.5. Activity of Applied Efe-AfpA on Expression of Dollar Spot Symptoms When Strong Creeping Red Fescue and Creeping Bentgrass Plants Were Inoculated with Clarireedia jacksonii in a Greenhouse Assay

**Figure 7:**
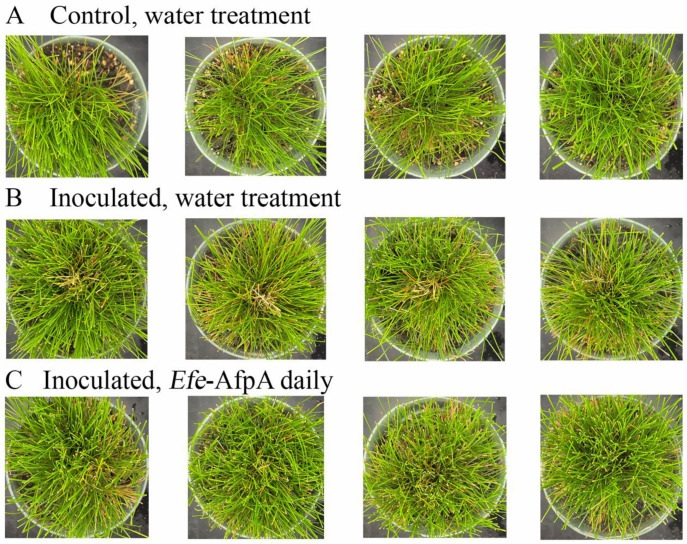
*Efe*-AfpA prevented severe symptoms of dollar spot disease when endophyte-free strong creeping red fescue plants were inoculated with an 8 mm plug of *C. jacksonii*. Plants were sprayed daily for 10 days with either water (**A**,**B**) or 100 μg mL^−1^ of *Efe*-AfpA (**C**). Photos within a row are replicates of the labeled treatment.

**Figure 8:**
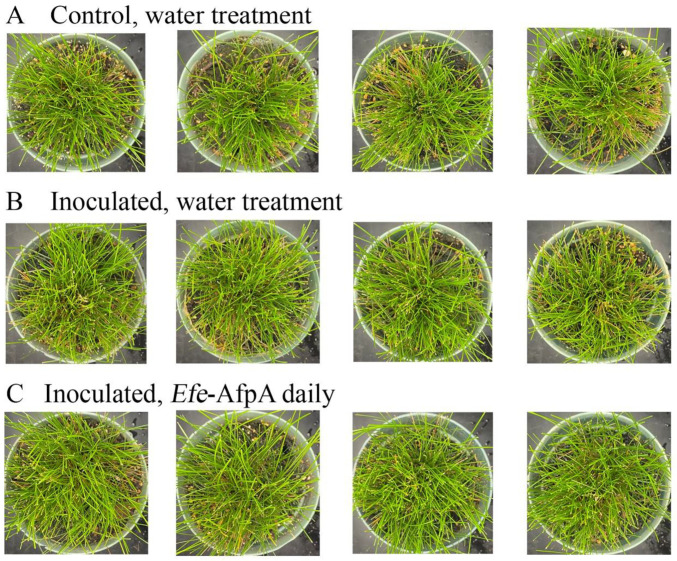
Endophyte infected strong creeping red fescue plants exhibited no dollar spot disease symptoms when inoculated with an 8 mm plug of *C. jacksonii*. Plants were sprayed daily for 10 days with either water (**A**,**B**) or 100 μg mL^−1^ of *Efe*-AfpA (**C**). Photos within a row are replicates of the labeled treatment.

As described above, in field studies, endophyte-infected strong creeping red fescue plants were resistant to dollar spot infection, whereas endophyte-free plants were susceptible to the disease [19]. *Efe*-AfpA is an abundant *E. festucae* protein in endophyte-infected plants and may be the reason for the disease resistance seen in endophyte-infected plants. it was therefore of interest to determine if application of the protein could protect endophyte-free plants from dollar spot caused by *C. jacksonii*. Purified *Efe*-AfpA was tested against *C. jacksonii* on endophyte-free and endophyte-infected strong creeping red fescue plants in greenhouse assays where an agar plug of *C. jacksonii* was used to inoculate the plants. Plants were sprayed with either water or a 100 μg mL^−1^ *Efe*-AfpA solution and the symptom expression of the controls and treated plants was compared. As seen in Figure 7, the *C. jacksonii* inoculated endophyte-free plants had severe disease symptoms, observed as necrotic tillers surrounding the point of inoculation in the center of plants. In contrast, the *Efe*-AfpA treated inoculated endophyte-free plants had only minor symptoms of dollar spot disease. The endophyte-infected plants inoculated with *C. jacksonii* had only minor disease symptoms (Figure 8), when compared with the endophyte-free plants, similar to what is seen in field studies [19]. These results support the hypothesis that *Efe*-AfpA is a major contributor to the dollar spot resistance seen in *E. festucae*-infected strong creeping red fescue plants.

**Figure 9:**
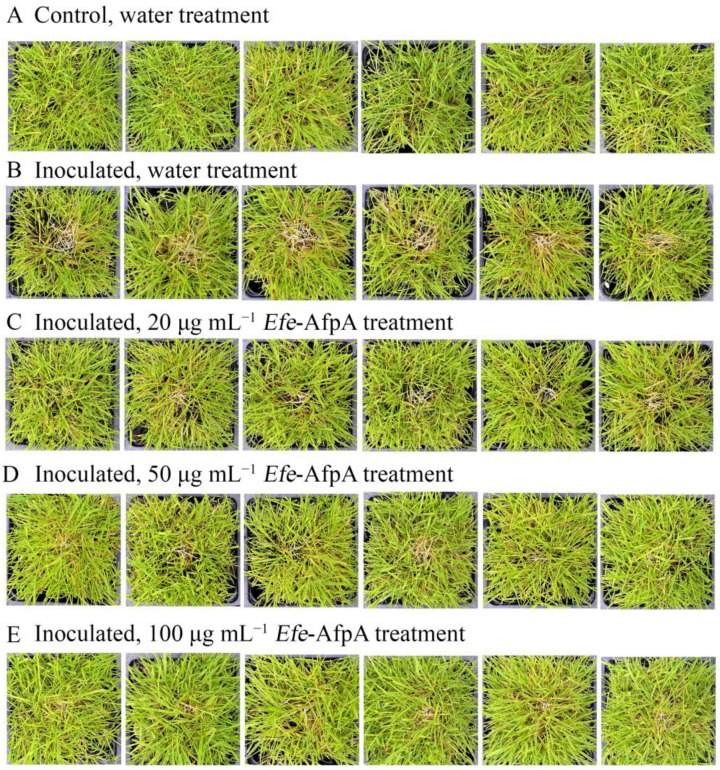
Effect of *Efe*-AfpA concentration on expression of dollar spot disease symptoms when creeping bentgrass cv. ‘Crenshaw’ plants were inoculated with an 8 mm plug of *C. jacksonii*. Plants were sprayed daily for 7 days with either water (**A**,**B**) or different concentrations of *Efe*-AfpA (**C**–**E**). Photos within a row are replicates of the labeled treatment.

Since dollar spot disease is a serious problem on creeping bentgrass, the ability of *Efe*-AfpA to protect creeping bentgrass plants from dollar spot caused by *C. jacksonii* was also determined in greenhouse assays. Plants were sprayed with either water or *Efe*-AfpA solutions and symptom expression of the control and treated plants was compared. Dollar spot symptoms on creeping bentgrass are characterized by necotic sunken areas of the turf [26] as seen in the inoculated water-treated plants (Figure 9). When sprayed daily for 7 days with either 20, 50 or 100 μg mL^−1^ *Efe*-AfpA, the plants had less severe disease symptoms at all concentrations, with 100 μg mL^−1^ providing the best protection.

**Figure 10:**
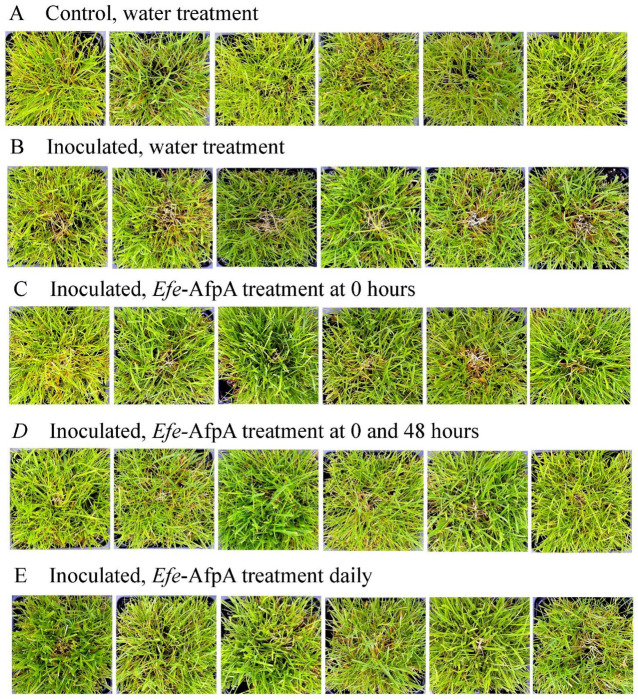
Effect of the number of treatments of *Efe*-AfpA applications on expression of dollar spot disease symptoms after creeping bentgrass cv. ‘Crenshaw’ plants were inoculated with an 8 mm plug of *C. jacksonii*. Plants were treated water (**A**,**B**) or with 100 μg mL ^−^^1^ of *Efe*-AfpA as indicated (**C**–**E**) and photographed 7 days post inoculation. Photos within a row are replicates of the labeled treatment.

To determine the effect of sequential applications on dollar spot severity, plants sprayed daily with 100 μg mL^−1^ *Efe*-AfpA were compared with plants sprayed only at the time of inoculation or at the time of inoculation and again at 48 h post-inoculation (Figure 10). The protection provided by two sprays was similar to that provided by seven daily applications.

### 3.6. Efe-AfpA Activity against Neurospora crassa Glucosylceramide Mutant Strains

PAF from *Pe. chrysogenum* and AFP from *A. giganteus* have been reported to require presence of the membrane sphingolipids glucosylceramide or Δ3-unsaturated glucosylceramide, respectively (Figure S4), in the susceptible target fungi for full antifungal activity [36,48]. Since *Efe*-AfpA and PAF have both similarities and differences in their activity against *N. crassa* and *C. jacksonii*, respectively, we compared their activities against some *N. crassa* membrane glucosylceramide mutants. The activities of PAF and *Efe*-AfpA were compared in the conidial growth assay with *N. crassa* glucosylceramide synthase (*gcs*) and 2-hydroxy fatty N-acyl-Δ3(E)-desaturase (sphingolipid Δ3(E)-desaturase), here designated *dtd* (delta three desaturase*)*, deletion mutants

In plate assays against wild type *N. crassa*, PAF and *Efe*-AfpA had similar levels of inhibition at concentrations of 3 μg mL^−1^ and above. At 25 μg mL^−1^ and above, there appeared to be complete inhibition of wild type *N. crassa* growth with both antifungal proteins. At the two lowest concentrations tested, PAF had higher levels of inhibition than *Efe*-AfpA (Figure S5). In plate assays, Huber et al. [36] reported reduced activity of PAF against the *N. crassa* Δ*gcs* mutant deficient in glucosylceramide relative to activity against the wild-type strain. The level of inhibitory activity was similar from 0.5 μM to 16 μM (3 μg mL^−1^ to 100 μg mL^−1^) [36]. Our results with PAF are similar, with similar levels of inhibition of the Δ*gcs* strain from 1.5 to 100 μg mL^−1^ (Figure S5). In contrast, the inhibitory activity of *Efe*-AfpA against the Δ*gcs* mutant was similar to its activity against the wild type strain (Figure S5), where *Efe*-AfpA exhibited complete inhibition at the higher concentrations. The activities of both PAF and *Efe*-AfpA against the Δ*dtd* mutant strain were similar to their activities against the wild-type strain (Figure S5).

**Figure 11:**
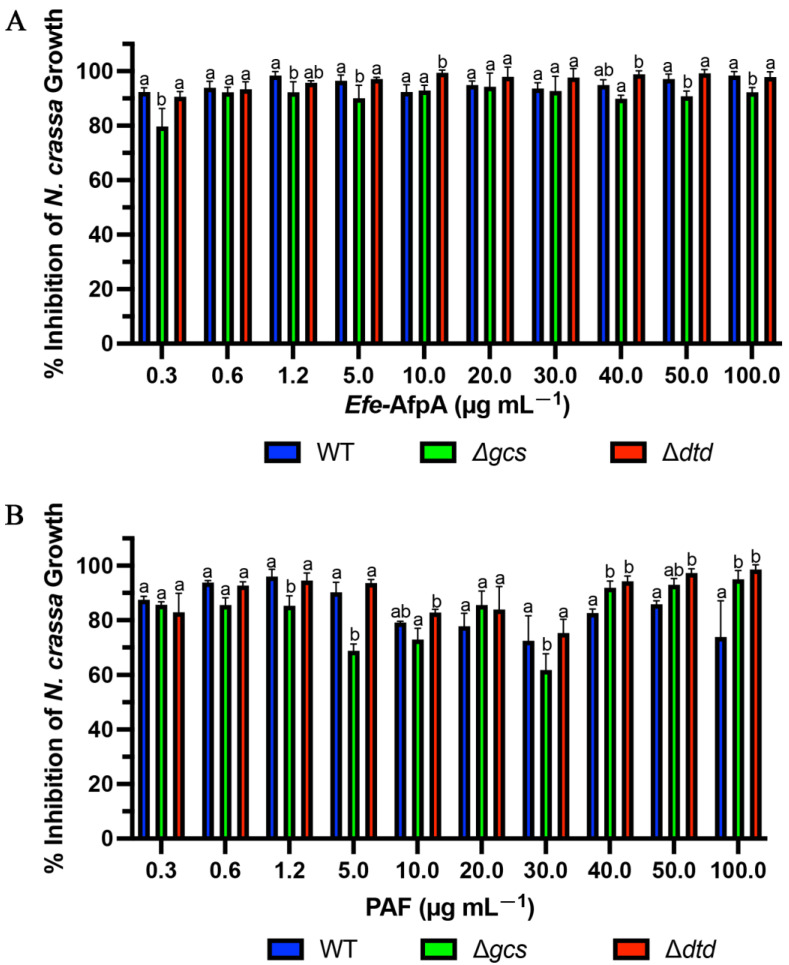
*Efe*-AfpA (**A**) and PAF (**B**) activity against *N. crassa* wild type and glucosylceramide pathway mutants. Increasing concentrations of the proteins were assayed for activity in the *N. crassa* growth assay with 1 × 10^4^ conidia mL^−1^. The data presented are the means and standard deviations of three replicates. For each concentration, columns with different letters indicate a significant difference in activity (*p* ≤ 0.05, two-way ANOVA).

In the quantitative 96-well microtitre plate liquid culture assays the inhibitory activity of PAF was reduced against the Δ*gcs* mutant strain relative to the wild-type strain at some, but not all, concentrations tested (Figure 11). The higher level of inhibition seen in Figure 11 relative to Figure 4 is because fewer *N. crassa* spores were used in Figure 11. Huber et al. [36] reported a lack of PAF inhibition of the growth of the Δ*gcs* mutant at 0.06 and 1 μM (approximately 0.37 and 6.2 µg mL^−1^). Here, at the similar PAF concentrations of 0.3 and 5 μg mL^−1^, there was a reduction in inhibition of growth of the Δ*gcs* mutant only at the higher concentration of 5 μg mL^−1^, but not the apparent complete loss of inhibition seen by Huber et al. [36]. Interestingly, at the higher concentrations of PAF, the Δ*gcs* mutant was more inhibited by PAF than the wild-type strain. At 1.2 and 5 μg mL^−1^, the Δ*gcs* mutant was also less inhibited by *Efe*-AfpA, but to a lesser extent than with PAF (Figure 11). There was no reduction in inhibition, relative to the wild-type strain, of the Δ*dtd* mutant by either PAF or *Efe*-AfpA.

With PAF, but not *Efe*-AfpA, the activity against the wild-type strain dropped between the concentrations 10 and 30 μg mL^−1^ and then rose at the higher concentrations. This drop in inhibitory activity of PAF against the wild-type *N. crassa* strain was reproducible, as it was seen in three independent assays. The explanation for this is unknown but does suggest there may be different modes of action at different concentrations. This decrease in inhibition (increased growth) can be seen in the accompanying microscopy (Figure S6).

From the 96 well liquid assays, the minimum inhibitory concentration (MIC) for *Efe*-AfpA and PAF against the wild-type and mutant *N. crassa* strains was determined (Table 3). The MIC is defined as the concentration needed for ≥90% inhibition. The low concentrations of 0.3 and 0.6 µg mL^−1^ for *Efe*-AfpA for all strains and for PAF for the wild type and Δ*dtd* strains are characteristic of antifungal proteins, as one of their hallmarks is high activity at low concentrations. The MIC of PAF for the Δ*gcs* mutant was much higher, at 40 µg mL^−1^. Although PAF does have inhibitory activity against the Δ*gcs* mutant, its reduced activity relative to the wild-type strain both in quantitative assays and in plate assays at some concentrations does support the proposal by Huber et al. [36] that membrane glucosylceramide in the target fungus is an important factor in PAF activity. The presence of glucosylceramides does not appear to be a major factor for *Efe*-AfpA activity.

In contrast to PAF, PAFB, a similar antifungal protein from *Pe. chrysogenum*, was found to have similar inhibition of the *N. crassa* wild type and glucosylceramide synthase mutant strains [36]). This difference was ascribed to a higher electrostatic affinity of PAFB to the anionic membrane surfaces [36]. *Efe*-AfpA also has a predicted higher net charge than PAF (Table 4), which may explain the differences in observed inhibition of the Δ*gcs* mutant strain relative to PAF.

**Figure 12:**
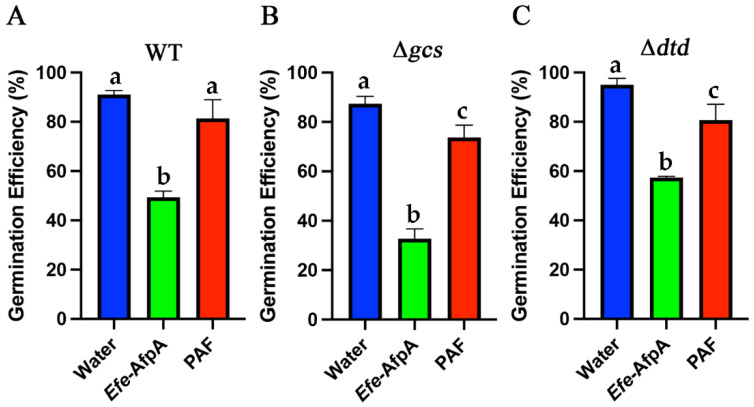
Effect of *Efe*-AfpA and PAF on conidia germination of *N. crassa* wild type (**A**) and glucosylceramide pathway mutants (**B**,**C**). Conidia (100 μL of 1 × 10^5^ conidia mL^−^^1^) were treated for six hours with either water or an antifungal protein at 10 μg mL^−^^1^. One-hundred conidia were counted as either germinated or ungerminated. The data presented are the means and standard deviations of three replicates. For each *N. crassa* strain, columns with different letters indicate a significant difference in activity (*p* ≤ 0.05, one-way ANOVA).

The comparison between PAF and *Efe*-AfpA for the inhibition of wild-type *N. crassa* growth in the 96-well liquid assay revealed that at concentrations between 10 and 100 μg mL^−1^ Efe-AfpA had significantly greater inhibitory activity (Figure S7). This effect was also seen with the two mutant strains at some concentrations. This phenomenon was further investigated by comparing conidial germination rates in the presence of 10 μg mL^−1^ PAF or *Efe*-AfpA (Figure 12). After 6 h of incubation, all three *N. crassa* strains had significantly fewer germinated conidia in the *Efe*-AfpA treated samples relative to the PAF treated samples when compared to the water treated control. The differences in activities of the two proteins seen in the 96-well liquid assay appears to be, in part, due to their differential effect on conidial germination.

### 3.7. Relationship of Efe-AfpA to Other Antifungal Proteins from Filamentous Fungi

**Figure 13:**
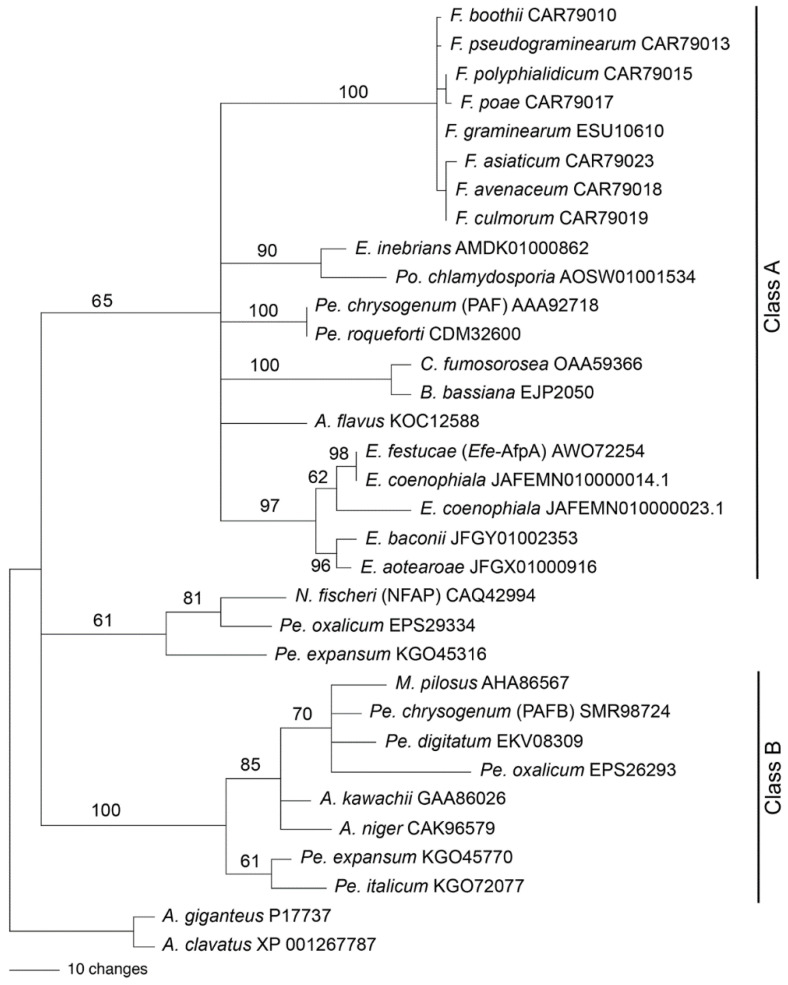
Rooted 50% majority rule maximum parsimony phylogenetic tree of the antifungal protein amino acid sequences. The *A. giganteus* and *A. clavatus* sequences were designated as the outgroups for rooting the tree. The numbers at the nodes are the bootstrap percentages based on 1000 replications. The tree was based upon 101 total characters, of which 14 were constant, 6 variable characters were parsimony uninformative, and 81 variable characters were parsimony informative. The NCBI accession numbers are given following the species names. The *Efe*-AfpA, PAF, NFAP, and PAFB sequences are identified in parentheses. The clades previously designated as Class A and Class B [49] are indicated. Genera abbreviations are F., *Fusarium*; E., *Epichloë*; Po., *Pochonia*; Pe., *Penicillium*; C., *Cordyceps*; B., *Beauveria*; A., *Aspergillus*; N., *Neosartorya*; M., *Monascus*.

We previously reported a phylogenetic analysis of antifungal proteins including *Efe*-AfpA [22]. Garrigues et al. [49] proposed a phylogenetic classification of the antifungal proteins. We therefore generated a new phylogenetic analysis to place *Efe*-AfpA into the antifungal protein classification scheme (Figure 13). The phylogeny reported here includes the sequences from Class A and Class B used previously [49], as well as some additional sequences. The Class C proteins were not included here since they are distinctly different than those of Classes A and B. *Efe*-AfpA, as well as the similar sequences from the other four *Epichloë* spp. that have antifungal protein genes, grouped with the Class A sequences.

Rooted phylogenetic trees can be informative regarding the evolutionary history of a protein, but can be problematic when the ancestral sequence is unknown or when there is horizontal gene transfer between unrelated species. Both of these issues are relevant with antifungal proteins. PAF from *Pe. chrysogenum* was placed in a clade of antifungal proteins from the Hypocreales rather than the Eurotiales, suggesting the possibility of horizontal gene transfer [22]. Large genomic regions containing the PAF gene sequence were found to be horizontally transferred among several cheese-associated *Penicillium* spp. [50,51]. The antifungal protein gene sequence from *E. inebrians* was more similar to that from *Pochonia chlamydosporia*, a parasite of nematode eggs, than to antifungal protein sequences from other *Epichloë* spp., suggesting its origin from horizontal gene transfer [20]. These issues make it difficult to choose a sequence with which to root the tree based on species phylogeny. The phylogenetic tree in Figure 13 was therefore rooted with the *A. giganteus* and *A. clavatus* sequences since those antifungal proteins are distinct from the rest, in that they have eight cysteines involved in four disulfide bonds [52,53] in contrast to six cysteines involved in three disulfide bonds in the other antifungal proteins used in the analysis.

The phylogenetic tree presented in Figure 13 is similar to that of Garrigues et al. [49] except in the placement of the sequences from *Neosartorya fischeri*, *Pe. oxalicum*, and *Pe. expansum*, which were previously grouped with the Class A proteins but here are not grouped with either of the two major clades. Hajdu et al. [54] also found that the NFAP-type antifungal proteins formed a clade separate from the Class A and Class B proteins. Here, all the Class B proteins are from fungal species within the Eurotiales whereas Class A contains species from both the Eurotiales and the Hypocreales.

### 3.8. Afp-A Genes in Epichloë spp.

We previously reported that most *Epichloë* spp. for which sequence data is available do not have a gene similar to *Efe*-*afpA* [20,22]. Since then, genome sequence data have become publicly available for two commercially important *Epichloë* spp., *E. festucae* var. *lolii* and *E. coenophiala*, which are endophytes of the turf and forage grasses perennial ryegrass and tall fescue, respectively. The *E. festucae* var. *lolii* AR5 genome (NCBI accession SRX1531627) [55] does not have a gene similar to *Efe*-afpA but the *E. coenophiala* genome does (NCBI accession JAFEMN010000000) [56].

Tall fescue is an important cool-season turf and forage grass used extensively worldwide. It is often infected with the endophyte *E. coenophiala*, which is a triparental hybrid fungus derived from the haploid species *E. festucae*, *E. typhina* subsp. *poae*, and a member of the *Lolium*-associated endophyte clade possibly derived from *E. baconii* [57,58]. The *E. coenophiala* genome sequence has two sequences similar to *Efe*-afpA, and these are included in the phylogenetic tree in Figure 13. At the amino acid level, the *E. coenophiala* AfpA sequence on scaffold 14 is 100% identical to that of *Efe*-AfpA and therefore likely derives from the *E. festucae* component of the *E. coenophiala* genome. The *E. coenophiala* AfpA sequence on scaffold 23 is more similar to that of *E. festucae* than to the AfpA sequence found in *E. baconii* and so cannot be unambiguously attributed to the *Lolium*-associated endophyte progenitor of *E. coenophiala*. However, the sequenced isolate of the other ancestral parent of *E. coenophiala*, *E. typhina* subsp. *poae*, does not have an AfpA gene in its genome [22].

Dinkins et al. [59] reported an extensive Illumina-based transcriptome comparison of endophyte-infected versus endophyte-free tall fescue. *E. coenophiala Efe*-*afpA*-like transcripts were represented, but at a low level. There were a total of 42 *Efe*-*afpA*-like reads in the endophyte-infected pseudostem samples out of a total of 1.3 million *E. coenophiala* reads, and 1 *Efe*-*afpA*-like read in the endophyte-infected leaf samples out of a total of 10,084 reads. Both *E. coenophiala* genes were represented among the reads. The low level of expression of the *E. coenophiala Efe*-*afpA*-like genes in endophyte-infected tall fescue is in stark contrast to the high level of expression in strong creeping red fescue infected with *E. festucae*, where *Efe*-*afpA* was among the most abundant fungal transcripts [22,60]. Tall fescue infected with *E. coenophiala* has not been reported to exhibit enhanced disease resistance in the field [19,20]. Therefore, it seems likely a high expression level of *afpA in planta*, as seen in strong creeping red fescue infected with *E. festucae*, is required for disease resistance. How expression of *Efe*-*afpA in planta* is regulated is unknown, but in strong creeping red fescue is 700-fold higher *in planta* than in culture [28].

## 4. Discussion

Here, we compared the activity of *Efe*-AfpA expressed in the prokaryote *E. coli* and in two eukaryotic systems, *Pi. pastoris* and *Pe. chrysogenum*. Active *Efe*-AfpA was obtained from all systems, with the *Pe. chrysogenum* system chosen as the most convenient. Purified *Efe*-AfpA clearly has antifungal activity, as shown here against *N. crassa* and *C. jacksonii*, and presumably also has antifungal activity in planta. Since application of purified *Efe*-AfpA was shown to protect endophyte-free strong creeping red fescue inoculated with *C. jacksonii* from developing severe symptoms of dollar spot, it seems likely that *Efe-*AfpA is the main factor in the observed field level resistance seen in endophyte-infected strong creeping red fescue. *Efe*-*afpA* knockout isolates of *E. festucae* were previously produced with the objective of directly assessing this presumption by inoculating the knockouts into endophyte-free strong creeping red fescue and determining if resistance to *C. jacksonii* was diminished relative to the wild type *E. festucae* strain [28]. However, the *Efe*-*afpA* knockout isolates were unable to infect the grass, whereas wild type and complemented isolates were able to infect. These results suggested that *Efe*-AfpA also affects the interaction between *E. festucae* and its host in addition to having antifungal activity. Similarly, additional roles for PAF in *Pe. chrysogenum* including conidiation and autophagy have been proposed [61,62].

*Efe-*AfpA expressed in *Pe. chrysogenum* was tested on creeping bentgrass plants inoculated with the dollar spot pathogen and was shown to be effective in reducing the severity of disease symptoms. Sapkota et al. [26] reviewed the economic importance of dollar spot disease on both cool-season and warm-season turfgrass species. Control of dollar spot disease on creeping bentgrass is a major problem for golf course managers and currently relies heavily on fungicide applications. Additionally, the dollar spot pathogen has developed reduced sensitivity or increased tolerance to many fungicide classes. Ongoing efforts to address this problem have focused on breeding tolerant cultivars and on developing and implementing improved best management protocols. The results presented here offer a potential additional/complementary approach for the management of dollar spot disease, the application of purified *Efe*-AfpA for use as an antifungal agent against this economically important plant pathogen.

The direct comparison of bacterial and eukaryotic expression systems reported here revealed the superiority of the *Pe. chrysogenum* system for producing *Efe*-AfpA, based on the quantity of protein produced and the ease of purification. However, the *E. coli* system was also effective at producing active *Efe*-AfpA. Bacterial expression systems are often used for eukaryotic proteins because of their ease of purification through affinity tags. Attempts at expression of antifungal proteins in *E. coli* have rarely been reported. An attempt at producing functional PAF from *P. chrysogenum* in bacteria was reported as unsuccessful, yielding only inactive protein, which was attributed to improper folding of the protein [63]. Similarly, expression of the antifungal protein from *Monascus pilosus* in *E. coli* resulted in a protein with 100-fold less activity than the native protein [64].

A factor that is likely important in expression of active *Efe*-AfpA in bacteria is the correct folding of the recombinant protein. Class A antifungal proteins, such as *Efe*-AfpA, all have six conserved cysteine residues involved in intramolecular disulfide bonds that likely contribute to the compact structure and stability of the proteins. The cysteine pairing in PAF from *P. chrysogenum* was determined by mass spectrometry of disulfide-bonded peptides [33]. The structure of PAF that is maintained by proper disulfide bonding is critical for the antifungal activity of the protein. The presence of glutathione, which resulted in a reduction in disulfide bonds, caused PAF to be maintained in the linear form and was inactive [33]. Production of disulfide-bonded proteins in *E. coli* has proved to be problematic because of the reducing environment of the cytoplasm. Here, *Efe*-AfpA was expressed in SHuffle cells, which are from an *E. coli* strain that has been engineered to constitutively express disulfide bond isomerase (DsbC) in the cytoplasm, which corrects disulfide bonding in incorrectly oxidized proteins, and in which thioredoxin reductase and glutathione reductase are deleted [45]. SHuffle cells were used previously to successfully produce functional small cysteine-rich effector proteins from fungal plant pathogens [65].

Another factor likely to be important in the production of active *Efe*-AfpA in bacteria is the use of the vector pETite, which adds a 6XHis-Sumo tag to the recombinant protein and allows the protein to be purified by metal affinity chromatography. The 107 amino acid 6XHIS-SUMO tag can then be cleaved precisely at the junction between the tag and the recombinant protein by SUMO protease, thereby leaving no extra amino acids on the target protein. Many bacterial expression systems inherently result in extra amino acids on the protein that often have no effect on protein activity. However, we found that even just six extra amino acids on the *Efe*-AfpA protein reduced the activity of the protein expressed in yeast [20]. A longer form of AFP from *A. giganteus* with six additional amino acids at the N-terminus had less activity than the fully mature form [66]. The reported previous attempts at expressing a similar antifungal protein in bacteria used systems that added 11 or 30 amino acids to the protein [63,64], which is common in many bacterial expression systems, and is presumably one reason those proteins had no or reduced activity. Apparently, the antifungal proteins cannot tolerate excessive extra amino acids, which results in loss of activity. Here, the N-terminal modified forms of *Efe*-AfpA, which added only one or two extra amino acids, were active. The combined use of the pETite vector, which has the precisely cleavable SUMO tag, and the SHuffle cells, which were designed to facilitate disulfide bonding, resulted in active *Efe*-AfpA. However, higher concentrations of the protein were required for the same level of activity, relative to the proteins expressed in *Pi. pastoris* or *Pe. chrysogenum*. Whether the lower activity when expressed in *E. coli* is due to the modified N-terminal amino acids or to less efficient disulfide bonding is not known. For *Efe*-AfpA the eukaryotic expression systems were clearly preferable for recovery of high levels of active protein.

The use of antifungal proteins as alternatives or complements to fungicides warrants further exploration. Here, we demonstrated the effectiveness of *Efe*-AfpA in protecting endophyte-free strong creeping red fescue and creeping bentgrass from developing severe symptoms of the important turfgrass disease dollar spot. Other antifungal proteins have also been reported to be effective in protecting plants from specific fungal pathogens. Direct application of an antifungal protein from *Pe. expansum*, PeAfpA, was successful in protecting orange fruit from infection by the post-harvest pathogen *Pe. digitatum* and tomato leaves from infection by *Botrytis cinerea* [67]. PAF applied to detached tomato leaves was protective against *B. cinerea* [68]. Gandia et al. [69] compared the effectiveness of three antifungal proteins from *Penicillium* spp. and NFAP2 from *N. fischeri* in delaying symptoms of *Penicillium* postharvest fruit decay. They reported some species-specific reductions in infections. Here, we also observed some species-specific effects. Although PAF and *Efe*-AfpA are similar proteins, their activity against *C. jacksonii* was quite different. Additionally, the comparison of PAF and *Efe-AfpA* in activity against the *N. crassa* Δgcs mutant revealed an apparent difference in the requirement for membrane glucosylceramide in the target fungus.

A challenge for the commercialization of antifungal proteins as alternatives or complements to fungicides is an efficient large-scale production system for the proteins. The *Pe. chrysogenum* system [31], as used here for *Efe*-AfpA, can yield 12 μg L^−1^ of culture and could be a viable system. Additionally, a plant-based transient expression system using tobacco mosaic virus has been developed and was successful in producing the *Pe. digitatum* antifungal protein AfpB in *Nicotiana benthamiana* [70]. Overall, the future development of *Efe*-AfpA, as well as other antifungal proteins, for use in combatting specific plant pathogenic fungi could provide new approaches to plant disease management and potentially help reduce fungicide inputs.

## Figures and Tables

**Table 1:** N-terminal modifications of mature *Efe*-AfpA expressed in *E. coli*.

Modification	Protein Designation	Protein Sequence
None	*Efe*-AfpA	ITYEGTCSRAKNECKYKNQNNKDTFVKCPSFANKKCTKDNAKCSFDSYSRAVTCH
I to A	A-*Efe*-AfpA	ATYEGTCSRAKNECKYKNQNNKDTFVKCPSFANKKCTKDNAKCSFDSYSRAVTCH
A to G	G-*Efe*-AfpA	GTYEGTCSRAKNECKYKNQNNKDTFVKCPSFANKKCTKDNAKCSFDSYSRAVTCH
A + G	GA-*Efe*-AfpA	GATYEGTCSRAKNECKYKNQNNKDTFVKCPSFANKKCTKDNAKCSFDSYSRAVTCH
A + GS	GSA-*Efe*-AfpA	GSATYEGTCSRAKNECKYKNQNNKDTFVKCPSFANKKCTKDNAKCSFDSYSRAVTCH

**Table 2:** Yield of purified *Efe*-AfpA from the different expression systems.

Expression System	Protein Yield (mg L^−1^ Culture)	Cultivation Time (Days) *
*Escherichia coli*	1	5
*Pichia pastoris*	3	9
*Penicillium chrysogenum*	12	9

* Time in days from plating to protein expression.

**Table 3:** Minimum inhibitory concentrations of *Efe*-AfpA and PAF against wild type and glucosylceramide mutant strains of *Neurospora crassa*.

	MIC (μg mL^−1^)	
*N. crassa* Strain	*Efe*-AfpA	PAF
WT	0.3	0.6
Δ*gcs*	0.6	40
Δ*dtd*	0.3	0.6

**Table 4:** Characteristics of the mature amino acid sequences of PAF, PAFB, and *Efe*-AfpA The numbers of charged amino acids in each protein are given.

Protein (Accession)	pI ^1^	Net Charge (pH 7.0) ^1^	Arg	Lys	His	Asp	Glu
PAF (AAA92718)	8.64	4.7	0	13	0	7	1
PAFB (SMR98724)	8.5	5.2	2	8	6	3	3
*Efe*-AfpA (AWO72254)	8.89	6.0	2	9	1	3	2

^1^ Determined by using Protein Calculator v3.4 (http://protcalc.sourceforge.net/). Website accessed 14 October 2022.

## Data Availability

All data supporting the findings of this study are available within the paper and within itsSupplementary Materials published online.

## Supplementary Materials

The following supporting information can be downloaded at: https://www.mdpi.com/article/10.3390/jof8101097/s1, Table S1: Sequences of oligonucleotide primers used in this study; Figure S1: SDS-PAGE of N-terminal modified Efe-AfpA proteins expressed in E. coli; Figure S2: Effect of Efe-AfpA on C. jacksonii mycelial growth; Figure S3: Effect of PAF on C. jacksonii mycelial growth; Figure S4: Final steps in the fungal sphingolipid biosynthetic pathway; Figure S5: Plate assays of effect of Efe-AfpA and PAF on growth of N. crassa wild type and glucosylceramide mutants; Figure S6: Microscopy of effect of Efe-AfpA and PAF on N. crassa wild type and glucosylceramide pathway mutants; Figure S7: Quantitative comparison of activity of Efe-AfpA and PAF against N. crassa wild type and the glucosylceramide mutants Δgcs and Δdtd.
