# Supplementary material for: The Epichloë festucae Antifungal Protein Efe-AfpA Protects Creeping Bentgrass (Agrostis stolonifera) from the Plant Pathogen Clarireedia jacksonii, the Causal Agent of Dollar Spot Disease
Source: J Fungi (Basel). 2022 Oct 18;8(10):1097. doi: 10.3390/jof8101097 (PMC9605492; doi:10.3390/jof8101097)
Supplement: Supplementary file 1 [file jof-08-01097-s001.zip › jof-1954325-supplementary.pdf]

The *Epichloe festucae* antifungal protein *Efe*-AfpA protects creeping bentgrass (*Agrostis stolonifera*) from the plant pathogen *Clarireedia jacksonii*, the causal agent of dollar spot disease

Patrick A. Fardella, Zipeng Tian, Bruce B. Clarke, Faith C. Belanger

Supplementary Materials

Table S1. Sequences of oligonucleotide primers used in this study.

	Forward primer, 5'-3'	Reverse primer, 5'-3'
**Preparation of N-terminal Ala expression vector**		
Replacing N-terminal Ile with Ala in pPICZalphaA	CGAGAAAAGAGCTACGTATG AAGGAACATG	AGAGATACCCCTTCTTCTTT AG
Cloning into pETite N-His SUMO Vector	CGCGAACAGATTGGAGGTGC TACGTATGAAGGAACATGT	GTGGCGGCCGCTCTATTACT AATGACACGTGACAGCTC
**Preparation of N-terminal Gly (Replacement) expression vector**		
Replacing N-terminal Ala with Gly in pETite N-His Sumo Vector	GATTGGAGGTGGAACGTATG AAGGAACATGTTC	TGTTCGCGGTGAGCCTCA
**Preparation of N-Terminal Gly (Insertion) expression vector**		
Inserting N-terminal Gly into N-terminal Ala pETite N-His SUMO Vector	GGAGCTACGTATGAAGGAACATG	ACCTCCAATCTGTTCGCG
**Preparation of N-Terminal Gly-Ser (Double Insertion) expression vector**		
Inserting N-Terminal Gly-Ser into N-terminal Ala pETite N-His Sumo Vector	GGATCCGCTACGTATGAAGGAACATG	ACCTCCAATCTGTTCGCG
**Sumo forward primer**	ATTCAAGCTGATCAGACCCCTGAA	
**Creating the pSK275:*Efe*-AfpA Vector**		
Amplifying pSK275 for production of the pSK275:*Efe*-AfpA		
	ATGGTCTCTGCGATCACCAG	TACAAACAAAGATGCAAGAG GCCATCTAGGCCATCAAGCTT ATCGATACCGTCGACCTCG
	AGCTTGATGGCCTAGATGGC	ATTCGTTTTTGGCTCTAGAA CATGTTCCTTCATACGTGAT TATGAAGGGCTTGAGATGAT
Amplify *Efe*-AfpA with pSK275 Overhang	ATCACGTATGAAGGAACATG	ACCAAAAACCATTAAATGCC CTGGTGATCGCAGAGACCATC TAATGACACGTGACAGCTC
**Sequencing Primers for pSK275:*Efe*-AfpAPAF to confirm *Efe*-AfpA Sequence**		

pSK275:*Efe*-AfpA F	TTCGACCTATAAGCATCCGCC	
pSK275:*Efe*-AfpA R		GCAGCAGTTTGATAGTTATCCCT
**Spore PCR to Confirm Positive *Efe*-AfpA Transformants**		
Efe-AfpA Forward/Reverse	ATCACGTATGAAGGAACATG	CTAATGACACGTGACAGCTC
**Spore PCR to Confirm *Efe*-AfpA Sequence**		
pSK275:*Efe*-AfpA F/R	TTCGACCTATAAGCATCCGCC	GCAGCAGTTTGATAGTTATCCCT
**Sequencing Primers for *Efe*-AfpA**		
pSK275:*Efe*-AfpA F	TTCGACCTATAAGCATCCGCC	
pSK275:*Efe*-AfpA R		GCAGCAGTTTGATAGTTATCCCT
pSK275:*Efe*-AfpA F 2	ATGCAAATCACCACAGTTGCC	
pSK275:*Efe*-AfpA R 2		CTAATGACACGTGACAGCTC
Efe-AfpA Forward 2	ATGCAAATCACCACAGTTGC	
Efe-AfpA Reverse		CTAATGACACGTGACAGCTC

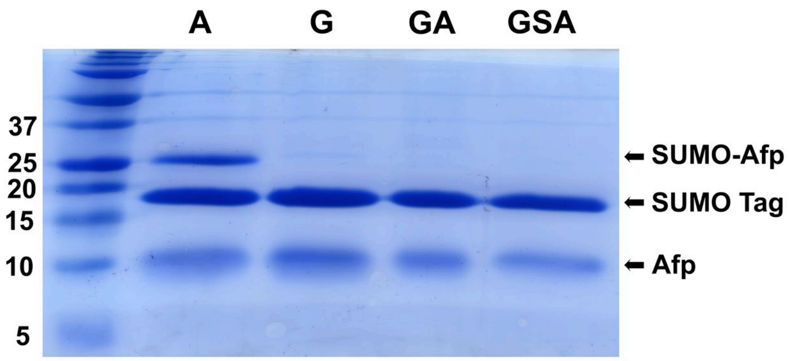

Figure S1. SDS-PAGE of N-terminal modified *Efe*-AfpA proteins expressed in *E. coli*. Proteins with the N-terminal modifications of A, G, GA, and GSA, were purified from *E. coli* lysates by binding to Talon Metal Affinity Resin. The purified proteins were then digested with the SUMO protease to remove the SUMO tag and the digested samples were subjected to SDS-PAGE. Arrows indicate the positions of the uncut SUMO-*Efe*-AfpA fusion protein, the released SUMO tag, and *Efe*-AfpA following digestion.

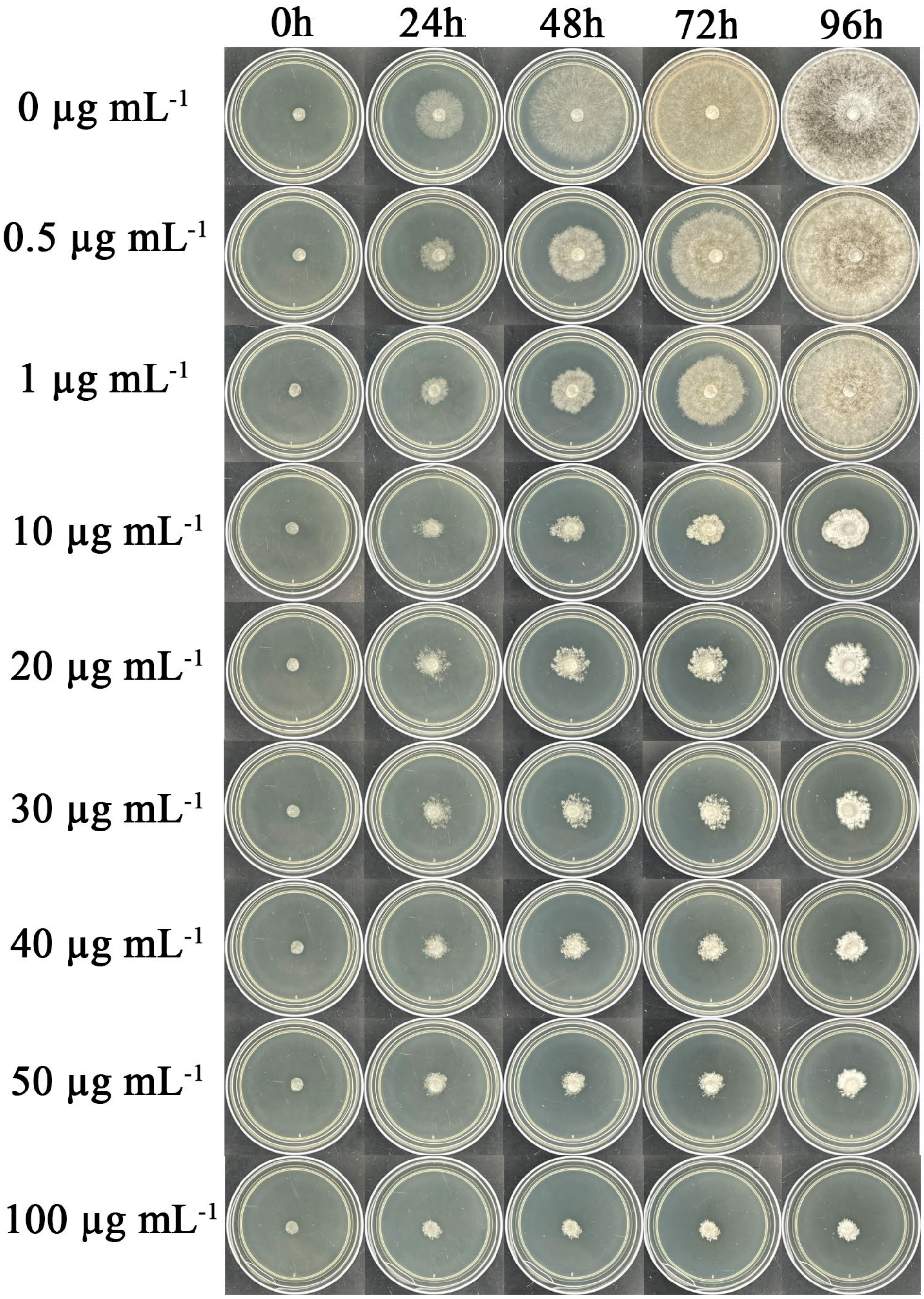

Figure S2. Effect of *Efe*-AfpA on *C. jacksonii* mycelial growth. *C. jacksonii* mycelial plugs were subcultured onto PDA plates amended with increasing concentrations of *Efe*-AfpA. The colony diameters were measured daily. The data presented are representative images of the three replicates.

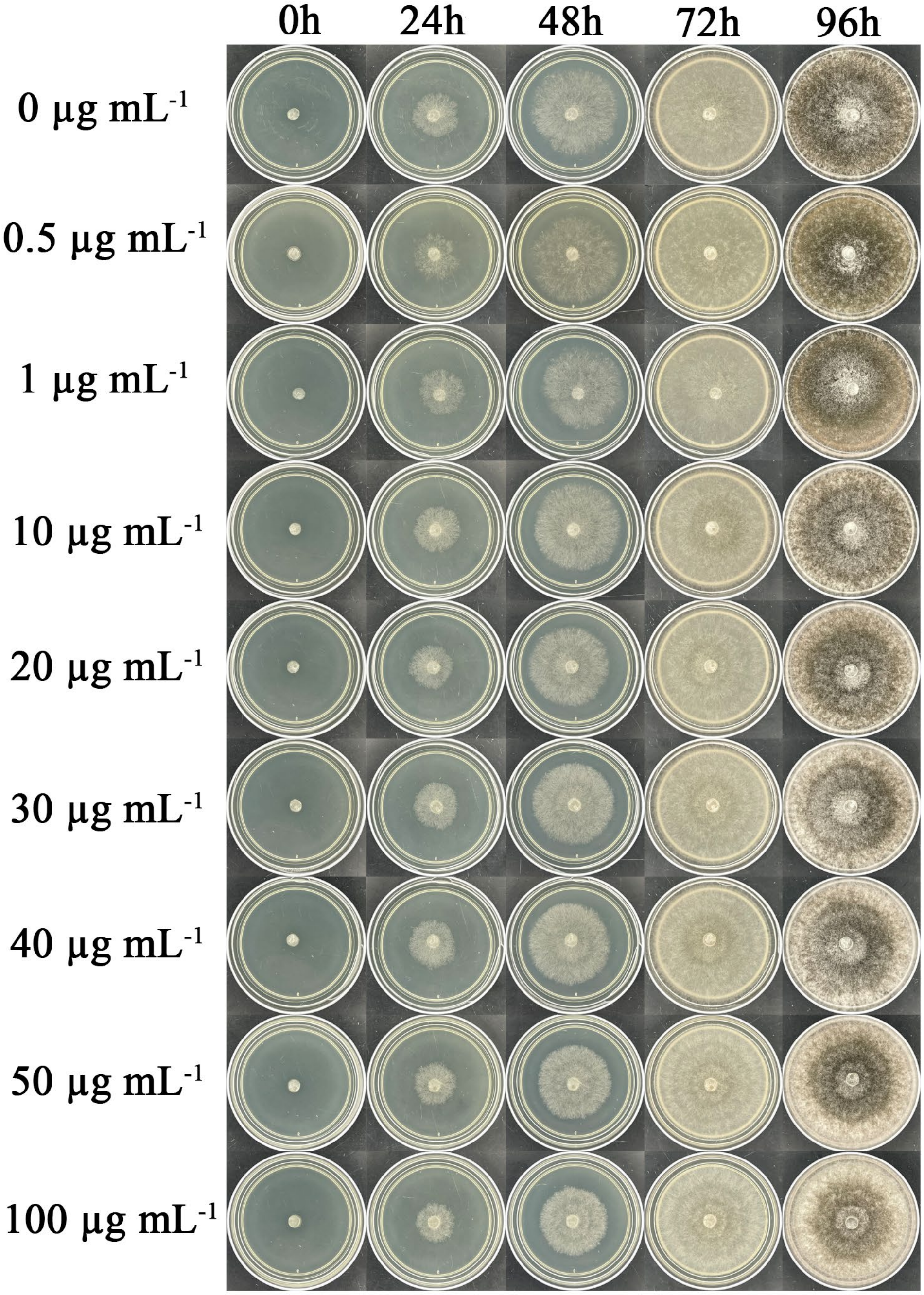

Figure S3. Effect of PAF on *C. jacksonii* mycelial growth. *C. jacksonii* mycelial plugs were subcultured onto PDA plates amended with increasing concentrations of PAF. The colony diameters were measured daily. The data presented are representative images of the three replicates.

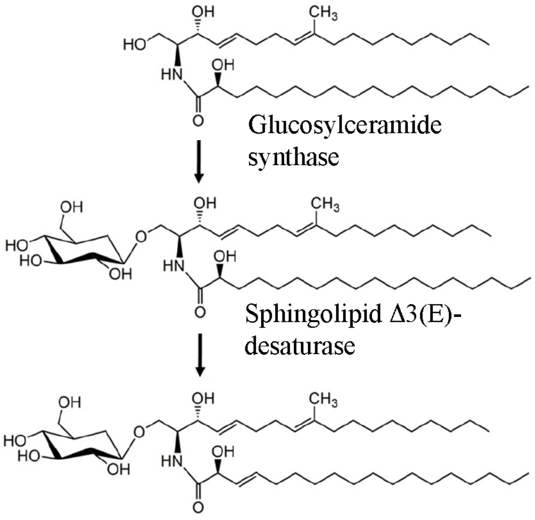

Figure S4. Final steps in the fungal sphingolipid biosynthetic pathway. Glucosylceramide synthase converts α-OH-◎4-Δ8-9, methyl-ceramide to α-OH-Δ4-◎8-9, methyl-glucosylceramide, which is converted to Δ3(E)-unsaturated glucosylceramide by sphingolipid Δ3(E)-desaturase.

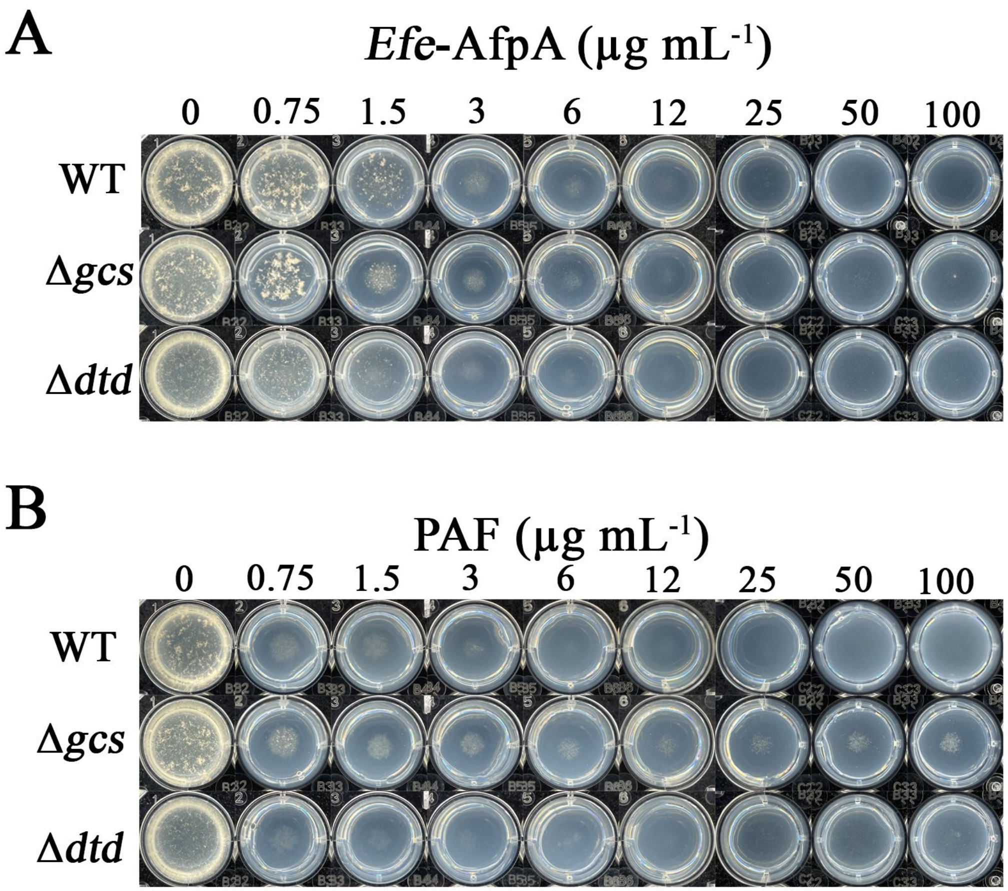

Figure S5. Plate assays of effect of *Efe*-AfpA (A) and PAF (B) on growth of *N. crassa* wild type and glucosylceramide mutants. Conidia were inoculated onto Vogel's media with increasing concentrations of either *Efe*-AfpA or PAF and photographed after 72 h at room temperature.

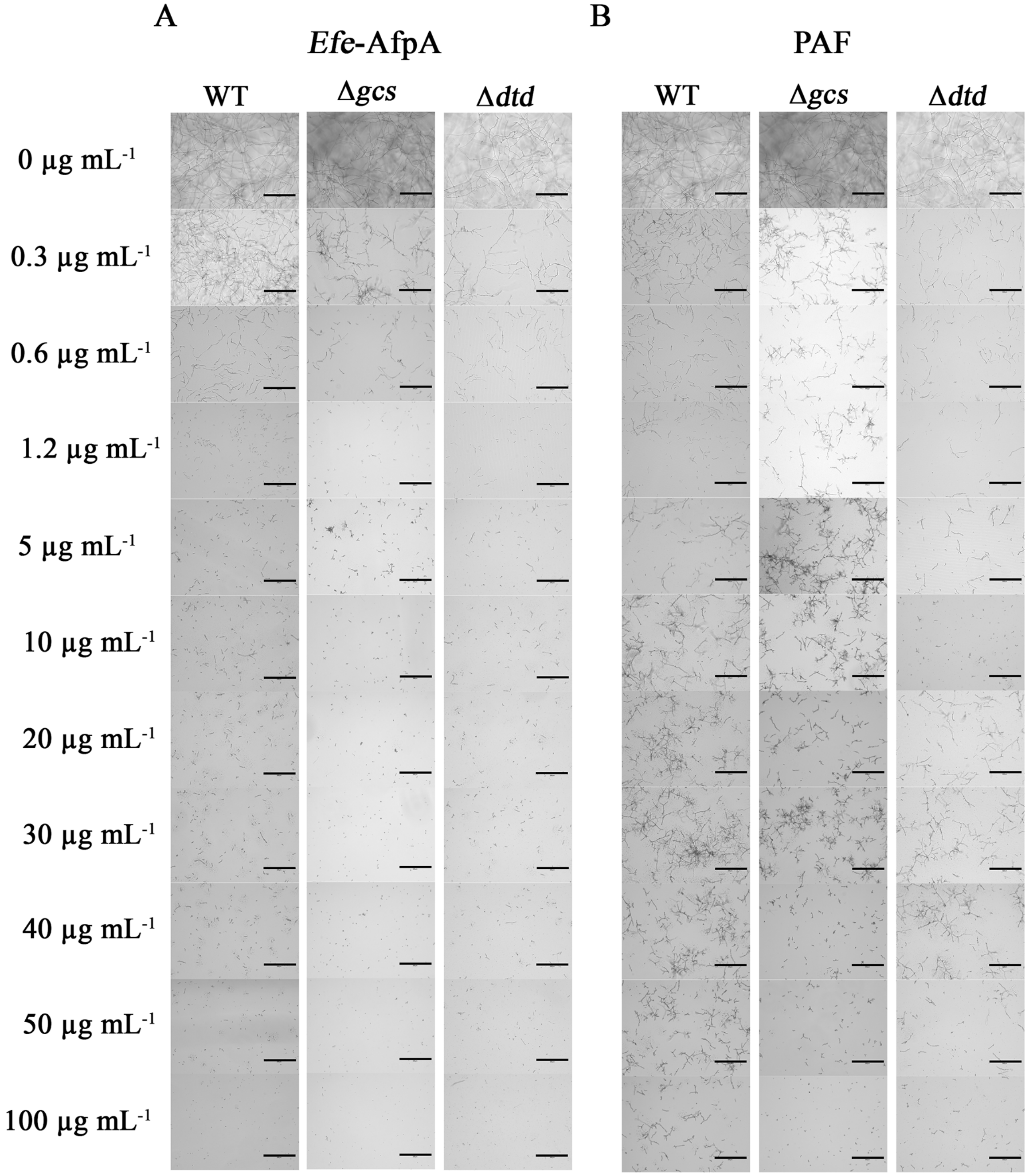

Figure S6. Microscopy of effect of *Efe*-AfpA (A) and PAF (B) on *N. crassa* wild type and glucosylceramide pathway mutants. Representative microscopy images of *N. crassa* conidia treated with increasing concentrations of *Efe*-AfpA or PAF. Bars are 300 µm.

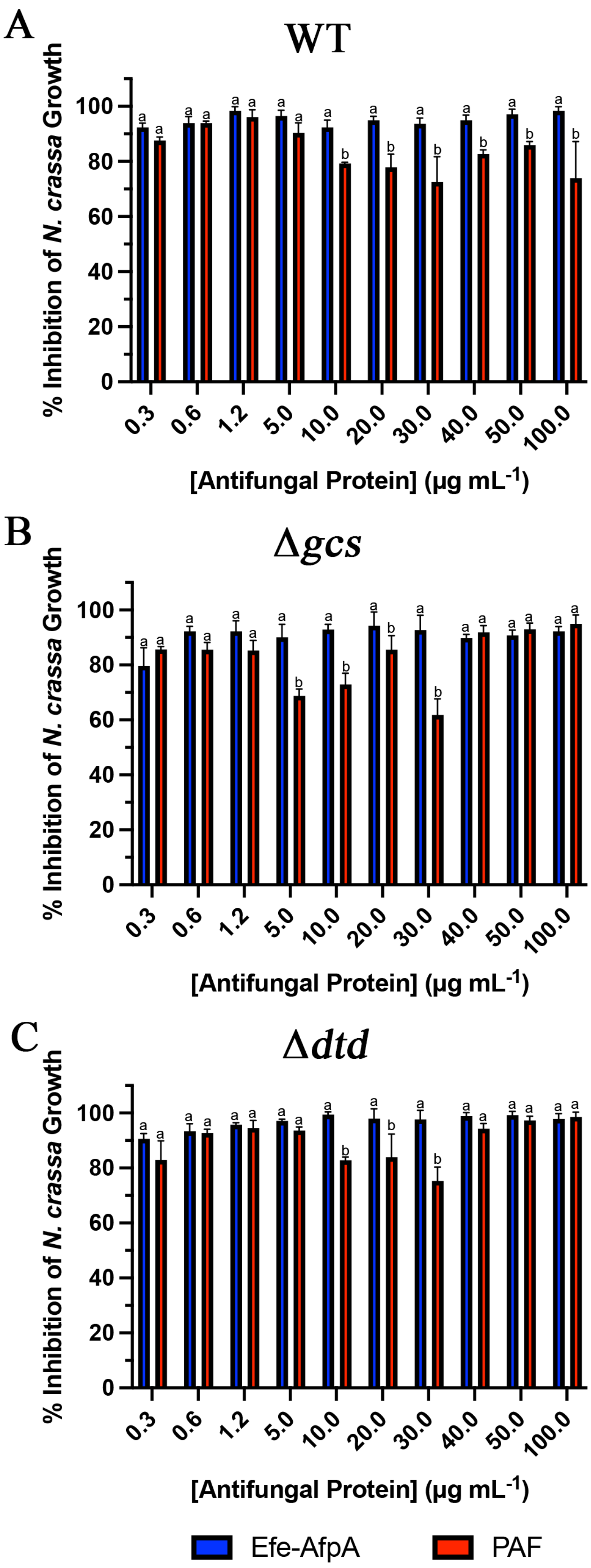
A
WT
B
Δgcs
C
Δdtd
% Inhibition of N. crassa Growth
[Antifungal Protein] (µg mL-1)
0.3
0.6
1.2
5.0
10.0
20.0
30.0
40.0
50.0
100.0
Efe-AfpA
PAF

Figure S7. Comparison of activity of *Efe*-AfpA and PAF against *N. crassa* wild type and the glucosylceramide mutants Δ*gcs* and Δ*dtd*. This is the same data used in Figure 11 but graphed to directly compare *Efe*-AfpA and PAF.
